# Antagonism between Prdm16 and Smad4 specifies the trajectory and progression of pancreatic cancer

**DOI:** 10.1083/jcb.202203036

**Published:** 2023-02-24

**Authors:** Eric Hurwitz, Parash Parajuli, Seval Ozkan, Celine Prunier, Thien Ly Nguyen, Deanna Campbell, Creighton Friend, Allyn Austin Bryan, Ting-Xuan Lu, Steven Christopher Smith, Mohammed Shawkat Razzaque, Keli Xu, Azeddine Atfi

**Affiliations:** 1 Department of Biochemistry and Molecular Biology, Massey Cancer Center, Virginia Commonwealth University, Richmond, VA, USA; 2 Cancer Institute, University of Mississippi Medical Centre, Jackson, MS, USA; 3 Sorbonne Université, Inserm, Centre de Recherche Saint-Antoine, CRSA, Paris, France; 4 Department of Pathology, Virginia Commonwealth University, Richmond, VA, USA; 5 Department of Pathology, Lake Erie College of Osteopathic Medicine, Erie, PA, USA

## Abstract

The transcription factor Prdm16 functions as a potent suppressor of transforming growth factor-beta (TGF-β) signaling, whose inactivation is deemed essential to the progression of pancreatic ductal adenocarcinoma (PDAC). Using the KrasG12D-based mouse model of human PDAC, we surprisingly found that ablating Prdm16 did not block but instead accelerated PDAC formation and progression, suggesting that Prdm16 might function as a tumor suppressor in this malignancy. Subsequent genetic experiments showed that ablating Prdm16 along with Smad4 resulted in a shift from a well-differentiated and confined neoplasm to a highly aggressive and metastatic disease, which was associated with a striking deviation in the trajectory of the premalignant lesions. Mechanistically, we found that Smad4 interacted with and recruited Prdm16 to repress its own expression, therefore pinpointing a model in which Prdm16 functions downstream of Smad4 to constrain the PDAC malignant phenotype. Collectively, these findings unveil an unprecedented antagonistic interaction between the tumor suppressors Smad4 and Prdm16 that functions to restrict PDAC progression and metastasis.

## Teaser

Pancreatic ductal adenocarcinoma (PDAC) is a deadly disease whose molecular etiology remains mostly enigmatic. By discovering an antagonistic relationship between the tumor suppressors Prdm16 and Smad4 in PDAC, this study paves the way for innovative frameworks with potential therapeutic implications.

## Introduction

Pancreatic ductal adenocarcinoma (PDAC) is the most aggressive type of pancreatic cancer, currently ranked as the fourth leading cause of cancer-related deaths in the United States (Connor and Gallinger, 2021; Hidalgo, 2010). Most of PDAC patients present with both locally invasive tumors and widespread metastasis, thus rendering ineffective the resection of the primary tumor as well as the applicability of the dismal therapeutic options available (Hidalgo, 2010; Stathis and Moore, 2010). Consequently, the outcome of PDAC patients remains extremely poor, with an overall 5-yr survival rate of less than 11%.

PDAC tumors emerge through three types of distinct precursor lesions called pancreatic intraepithelial neoplasia (PanIN), intraductal papillary mucinous neoplasia (IPMN), and mucinous cystic neoplasia (MCN), respectively (Connor and Gallinger, 2021; Yonezawa et al., 2008). These early-stage lesions harbor various genetic alterations, the earliest and most pervasive of which are activating mutations in KRAS, occurring in ∼90% of PDAC tumors (Hayashi et al., 2021). The current model posits that mutational activation of KRAS represents an essential initiating event, whereas subsequent accumulation of inactivating mutations in the tumor suppressor genes p16INK4a, SMAD4, and TP53 is necessary for PDAC to progress and metastasize (Hayashi et al., 2021; Iacobuzio-Donahue, 2012). Significant efforts have been made over the past two decades to create genetically engineered mouse models (GEMMs) that faithfully recapitulate the prominent features of human PDAC. For instance, pancreas-specific expression of KrasG12D in mice is sufficient to initiate PanINs, which occasionally progress into invasive PDAC following a long latency period, supporting the general notion that oncogenic activation of KRAS represents the main initiating genetic event in PDAC (Buscail et al., 2020; Hingorani et al., 2005; Tuveson et al., 2004; Westphalen and Olive, 2012). Concomitant expression of KrasG12D and deletion of any of the three cardinal tumor suppressors, e.g., Trp53, p16Ink4a, Smad4, accelerate PDAC progression, though the nature and final outcome of the tumors might differ. Indeed, while mice with the combined expression of KrasG12D and deletion of Trp53 (KPC) or p16Ink4a (KIC) develop PanINs that progress very rapidly to highly aggressive and metastatic PDAC, mice with the combined expression of KrasG12D and deletion of Smad4 (KSC) develop mostly IPMNs, which also progress to invasive PDAC, but the terminal disease develops with a much slower onset and manifests an attenuated metastatic phenotype (Bardeesy et al., 2006a; Bardeesy et al., 2006b; Hingorani et al., 2005; Izeradjene et al., 2007). Other examples of PDAC GEMMs include KTβC mice, which harbor KrasG12D and deletion of the transforming growth factor-beta (TGF-β) type II receptor (TβRII) gene, the latter being inactivated by mutations or deletions in 4% of PDAC (Iacobuzio-Donahue, 2012; Ijichi et al., 2006).

TGF-β signaling regulates a wide array of biological processes vital for normal cell growth, function, and homeostasis (David and Massagué, 2018; Massagué, 2008). TGF-β initiates signaling by inducing the assembly of a receptor complex composed of two types of transmembrane serine/threonine kinases called TβRI and TβRII. In that complex, the constitutive kinase of TβRII phosphorylates and activates the kinase activity of TβRI, which then propagates the signal to the nucleus through phosphorylation of Smad2 and Smad3 (David and Massagué, 2018; Feng and Derynck, 2005; Massagué et al., 2005). Once phosphorylated, Smad2 or Smad3 associates with Smad4, and the two complexes accumulate in the nucleus to regulate the expression of TGF-β target genes through cooperative interactions with transcriptional cofactors or corepressors (David and Massagué, 2018; Feng and Derynck, 2005; Massagué, 2008; Massagué et al., 2005).

Because of the widespread roles of TGF-β signaling in cellular functions, there must be multiple levels of positive and negative regulations to fine-tune initiation, magnitude, or termination of the response depending on the cell type or physiological context. One example of the mechanisms that limit TGF-β signaling involves the transcription factor PR domain containing 16 (Prdm16). Upon accumulation in the nucleus, the Smad3/Smad4 complex associates with the general transcriptional co-activators CBP and p300 to activate transcription of TGF-β target genes (David and Massagué, 2018; Feng and Derynck, 2005; Massagué, 2008; Massagué et al., 2005). Conversely, the Smad complex can also associate with Prdm16 and its partner c-Ski, which leads to the recruitment of general transcriptional corepressor complexes containing histone deacetylases and concomitant displacement of CBP and p300, thereby resulting in transcriptional repression (Takahata et al., 2009).

In addition to its function as a suppressor of TGF-β signaling, Prdm16 has been shown to play key roles in a number of biological processes, including differentiation of brown fat and specification of hematopoietic and neuronal stem cell fate (Chi and Cohen, 2016; Seale et al., 2007; Shimada et al., 2017). Prdm16 possesses a methyltransferase activity that catalyzes the methylation of Lysine-9 on histone-3 (H3K9), a mark associated with heterochromatin formation and gene expression (Jambhekar et al., 2019; Pinheiro et al., 2012). Recently, Prdm16 loss-of-function has been shown to play an instrumental role in leukemia driven by the MLL fusion oncoprotein (Zhou et al., 2016). Because the MLL gene encodes a histone-3 Lysine-4 (H3K4) methyltransferase that is critical in promoting gene expression during hematopoiesis (Xue et al., 2019), it has been postulated that Prdm16 might suppress leukemia pathogenesis owing to its ability to drive heterochromatin formation (Pinheiro et al., 2012; Zhou et al., 2016). At present, whether Prdm16 has any role in cancer pathogenesis and progression that is linked to its function in TGF-β signaling is still unknown. Here, we combined several orthogonal approaches and GEMMs to demonstrate that Prdm16 functions downstream of Smad4 to suppress PDAC progression and metastasis. As such, our findings unveil a previously uncharacterized mechanism that orchestrates Prdm16 tumor-suppressive function, and further shed new insights into the molecular etiology of PDAC, a fatal disease for which no effective therapeutics are currently available.

## Results

### Transient expression of Prdm16 during PDAC progression

**Figure 1.**
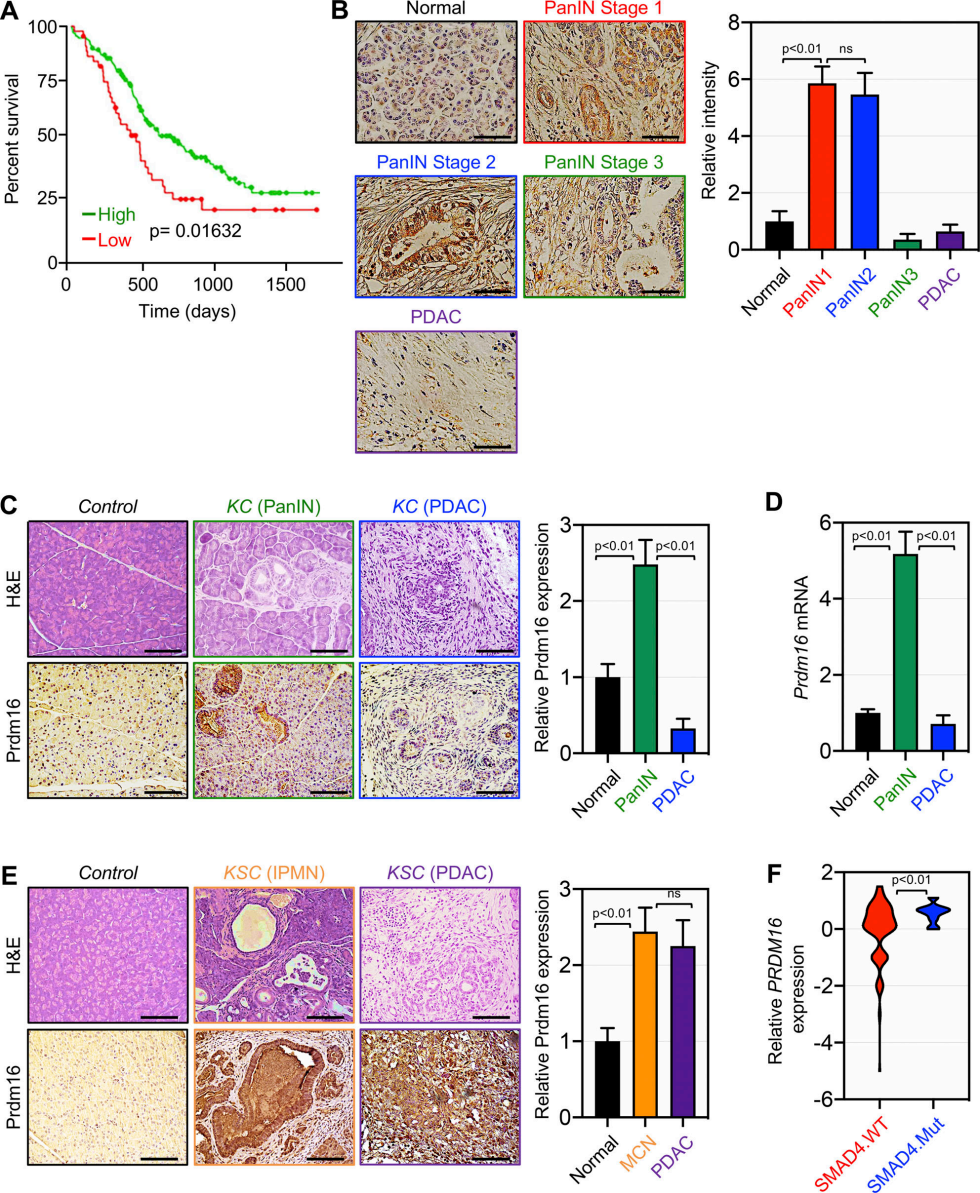
**Transient expression of Prdm16 during PDAC progression. (A)** Kaplan-Meier survival of PDAC patients based on high versus low PRDM16 expression was conducted using the TCGA dataset. Statistical power was assessed by log-rank test for significance. **(B)** Prdm16 protein expression was analyzed by IHC using human PDAC TMAs containing both normal tissues and PanIN/PDAC lesions (*n* = 152). Representative pictures of normal, PanIN, and PDAC areas are shown. Scale bars: 50 μm (left). Relative Prdm16 expression in normal, PanIN, and PDAC areas (right). Data are expressed as mean ± SEM. **(C)** FFPE pancreatic sections from 4-mo-old control and KC mice (*n* = 31 to 33) were stained with H&E or immunostained with anti-Prdm16 antibody and subjected to IHC. Representative pictures of normal, PanIN, and PDAC areas are shown. Scale bars: 50 μm (left). Relative Prdm16 expression in normal, PanIN, and PDAC areas are shown (right). Data are expressed as mean ± SEM. **(D)** Expression of Prdm16 mRNA in pancreas from 3-mo-old control or KC mice (*n* = 6) with PanIN or 10-mo-old KC mice with terminal PDAC was analyzed by qRT-PCR. Data are expressed as mean ± SEM. **(E)** FFPE pancreatic sections from 4-mo-old control and KSC mice (*n* = 31–45) were stained with H&E or immunostained with anti-Prdm16 antibody and subjected to IHC. Representative pictures of normal, IPMN and PDAC areas are shown. Scale bars: 50 μm (left). Relative Prdm16 expression in normal, IPMN and PDAC areas are shown (right). Data are expressed as mean ± SEM. **(F)** Relative expression of PRDM16 in human samples with wild-type or mutated SMAD4 was conducted using the TCGA dataset. Data are presented as a violin plot. Statistical power in B–F was assessed by a two-tailed, unpaired Mann–Whitney test.

**Figure S1.**
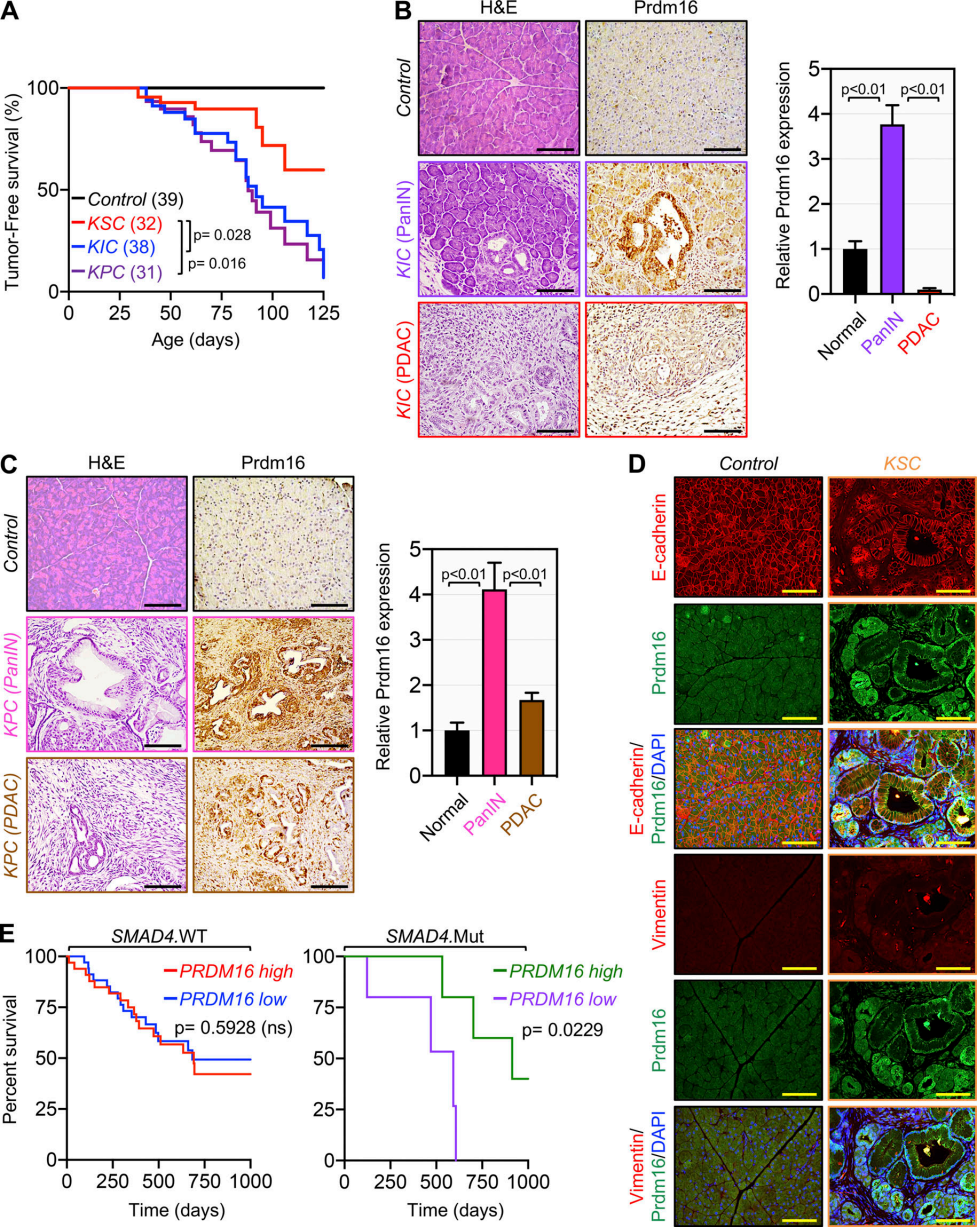
**Transient expression of Prdm16 during PDAC progression. (A)** Kaplan-Meier survival analysis of control, KSC, KIC, and KPC mice (*n* = 31–39). Statistical power was assessed by a log-rank test for significance. **(B)** FFPE pancreatic sections from 4-mo-old control and KIC mice (*n* = 38–39) were stained with H&E or immunostained with anti-Prdm16 antibody and subjected to IHC. Representative pictures of normal, PanIN and PDAC areas are shown. Scale bars: 50 μm (left). Relative Prdm16 expression in normal tissue and PanIN or PDAC lesions are shown (right). Data are expressed as mean ± SEM. **(C)** FFPE pancreatic sections from 3-mo-old control and KPC mice (*n* = 31–39) were stained with H&E or immunostained with anti-Prdm16 antibody and subjected to IHC. Representative pictures of normal, PanIN, and PDAC areas are shown. Scale bars: 50 μm (left). Relative Prdm16 expression in normal tissue and PanIN or PDAC lesions are shown (right). Data are expressed as mean ± SEM. **(D)** FFPE pancreatic sections from control and KSC mice (*n* = 31–45) were subjected to co-IF using antibodies to Prdm16 and E-cadherin or vimentin. Representative pictures of normal tissue and IPMN or PDAC areas are shown. Scale bars: 50 μm. **(E)** Kaplan-Meier survival analysis of patients with wild-type or mutated SMAD4 based on high versus low PRDM16 expression was conducted using the TCGA dataset. Statistical power was assessed by log-rank test for significance. Statistical power in B and C were assessed by a two-tailed, unpaired Mann–Whitney test.

**Figure S2.**
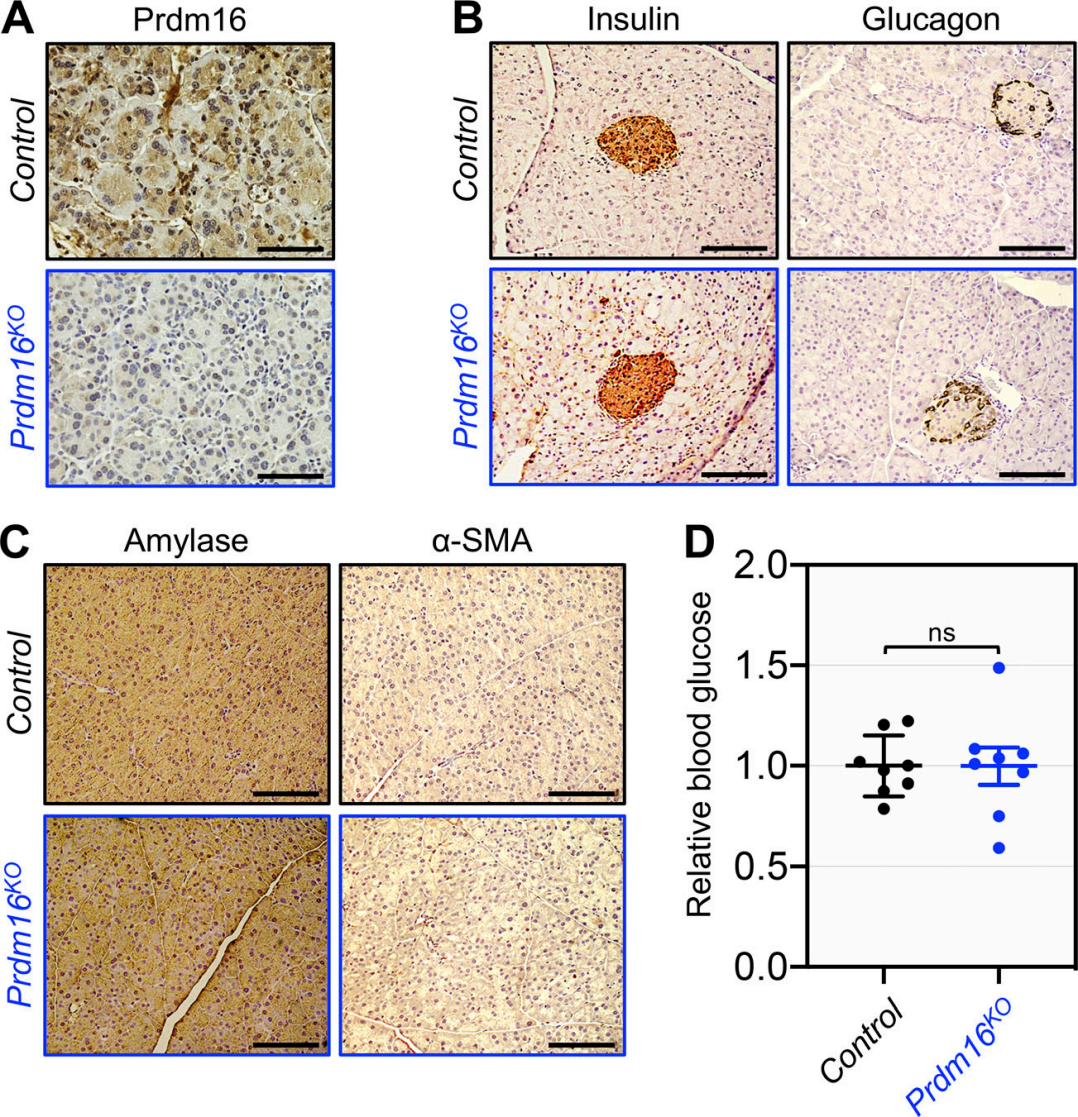
**Prdm16KO mice display normal pancreatic endocrine function. (A)** FFPE pancreatic sections from 15-wk-old control and Prdm16KO mice (*n* = 8–31) were immunostained with anti-Prdm16 antibody and subjected to IHC. Scale bars: 50 μm. **(B)** FFPE pancreatic sections from 15-wk-old control and Prdm16KO mice (*n* = 8–31) were immunoreacted with antibodies to insulin or glucagon and subjected to IHC. Scale bars: 50 μm. **(C)** FFPE pancreatic sections from 15-wk-old control and Prdm16KO mice were subjected to IHC using antibodies to amylase or α-SMA. Scale bars: 50 μm. **(D)** Blood glucose of 15-wk-old control or Prdm16KO mice (*n* = 8). Statistical power was assessed by a two-tailed, unpaired Mann–Whitney test.

To explore the possible involvement of Prdm16 in PDAC, we conducted Kaplan-Meier analysis using The Cancer Genome Atlas (TCGA) dataset. As shown in Fig. 1 A, low PRDM16 expression is associated with poor survival, providing an initial hint that Prdm16 might function as a tumor suppressor in PDAC. To substantiate this finding, we analyzed Prdm16 expression by immunohistochemistry (IHC) using large human tissue microarrays (TMAs) comprising samples with tumor lesions at various stages (e.g., PanIN1, PanIN2, PanIN3, PDAC) and normal tissues. Using a highly specific antibody to Prdm16 (see Fig. S2 A), we detected Prdm16 expression in both cancerous lesions and stromal areas (Fig. 1 B). Interestingly, Prdm16 expression appeared to fluctuate significantly during PDAC progression, commencing with a relatively low level in normal tissue, then rising in early PanINs, and finally declining to the background level in invasive PDAC (Fig. 1 B). Although this finding fits well with the notion that Prdm16 expression might be downregulated because of the accumulation of late genetic or epigenetic alterations, it did not shed light on the mechanisms leading to its transient expression during PDAC progression. To address this issue rigorously, we sought to utilize GEMMs that faithfully recapitulate the human PDAC in a uniform genetic background (Bardeesy et al., 2006a; Bardeesy et al., 2006b; Hingorani et al., 2005; Izeradjene et al., 2007; Tuveson et al., 2004). We initially utilized mice with pancreas-specific expression of KrasG12D alone (KC) and detected transient expression of the Prdm16 protein during PDAC progression, similar to what was observed in human PDAC, being relatively high in PanINs and very modest to low in normal tissue and invasive PDAC (Fig. 1 C). Confirmation of this result was obtained by comparative qRT-PCR experiments using cohorts of KC mice at the age of 3 mo when they experience mostly PanINs and 10 mo when they display visible signs of terminal PDAC (Fig. 1 D; Parajuli et al., 2020; Parajuli et al., 2019). To understand this phenomenon more deeply, we generated mice with KrasG12D together with deletion of Trp53 (KPC), p16Ink4a (KIC), or Smad4 (KSC). Noteworthy, we found that KSC mice had a longer survival rate than KIC and KPC mice, while the two latter had almost similar survival (Fig. S1 A). With regard to Prdm16 expression, we found that KIC and KPC mice behaved similarly to KC mice (Fig. S1, B and C), suggesting that transient expression of Prdm16 might take place even under the presence of the most common and aggressive genetic alterations that facilitate PDAC progression (Hayashi et al., 2021; Iacobuzio-Donahue, 2012). But most appealing was the fact that Prdm16 expression in KSC mice did not follow this transient pattern of Prdm16 expression, increasing markedly in IPMN lesions but thereafter remaining constant in PDAC lesions (Fig. 1 E), suggesting that Smad4 might influence Prdm16 expression during the progression from IPMN to full-blown PDAC. Co-immunofluorescence assays using anti-Prdm16 antibody together with antibodies to E-cadherin (epithelial marker) or vimentin (mesenchymal marker) showed that Prdm16 expression remained very high in E-cadherin + cells as compared to vimentin + cells (Fig. S1 D). Consistent with these findings, we found that patients with low expression of Prdm16 had the worst survival if they carry SMAD4 mutations (Fig. S1 E). Moreover, interrogating the TCGA dataset revealed that samples with deleterious genetic alterations in SMAD4 display higher expression of PRDM16 as compared to samples with wild-type SMAD4 (Fig. 1 F). As such, these data hint at the existence of an antagonistic association between Smad4 and Prdm16 during PDAC progression; we will return to this notion later.

### Prdm16 accelerates KrasG12D-driven PDAC

**Figure 2.**
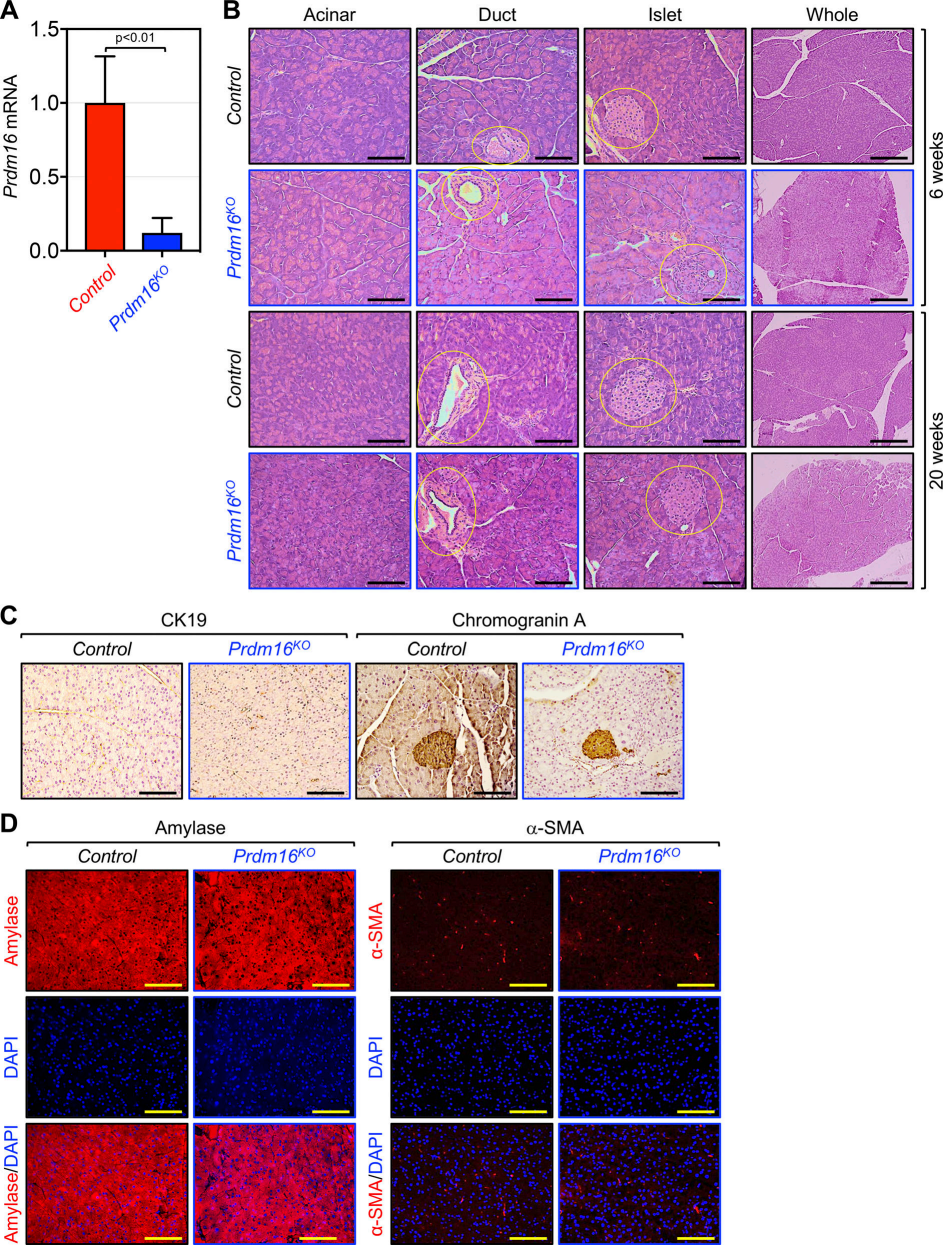
**Prdm16 inactivation did not affect pancreas histology or function. (A)** Prdm16 mRNA expression in 3-mo-old control and Prdm16KO mice was measured by qRT-PCR (*n* = 6). Data are expressed as mean ± SEM, and statistical power was assessed by a two-tailed, unpaired *t* test. **(B)** FFPE pancreatic sections from control or Prdm16KO mice (*n* = 8) at 6 or 20 wk-old were stained with H&E. Scale bars: 200 μm for “whole” pictures and 50 μm for all other pictures. **(C)** FFPE pancreatic sections from 15-wk-old control and Prdm16KO mice (*n* = 8 to 31) were immunostained with anti-CK19 or anti-Chromogranin A antibody and subjected to IHC. Scale bars: 50 μm. **(D)** FFPE pancreatic sections from 15-wk-old control and Prdm16KO mice (*n* = 8) were immunoreacted with antibodies to amylase or α-SMA before being subjected to immunofluorescence. Scale bars: 50 μm.

The aforementioned data prompted us to investigate whether Prdm16 could contribute to PDAC initiation, progression, or both. To do so, we generated mice with pancreas-specific deletion of Prdm16 (Prdm16KO) by crossing mice bearing a floxed allele of Prdm16 with Pdx1-Cre mice, which express Cre recombinase in all pancreatic progenitor cells that give rise to ductal, acinar, and islets compartments very early (E8.5) during development (Gu et al., 2003). Prdm16KO mice were born with the normal Mendelian frequency, develop normally without any signs of anatomic abnormalities, and were fertile. Effective deletion of Prdm16 in the pancreatic epithelium was confirmed by RT-PCR and IHC (Fig. 2 A and Fig. S2 A). To investigate whether Prdm16 deficiency could affect pancreas histology or function, we conducted a comprehensive analysis of pancreatic sections either by hematoxylin and eosin (H&E) staining, IHC or immunofluorescence (IF) encompassing all major tissue compartments, including duct (cytokeratin 19, CK19), acini (amylase), stroma (α-SMA), and islet (insulin, glucagon, chromogranin-A). We were not able to detect any noticeable changes in all three compartments irrespective of the age of mice analyzed (Fig. 2, B–D; and Fig. S2, B and C). Congruently, there was also no difference in fasting blood glucose between wild-type and Prdm16KO mice (Fig. S2 D). Thus, inactivation of Prdm16 throughout embryonic development and postnatal life was insufficient to perturb pancreas homeostasis or drive sporadic pancreatic cancers.

**Figure 3.**
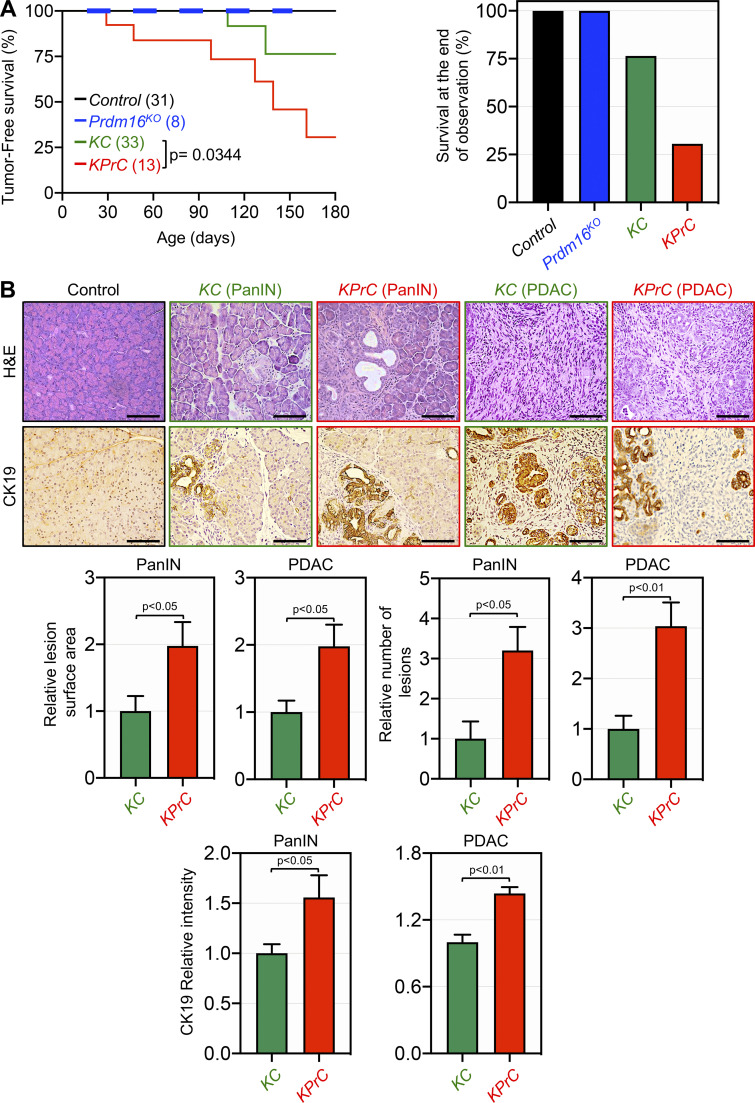
**Prdm16 inactivation accelerates KrasG12D-driven PDAC. (A)** Kaplan-Meier survival of control, Prdm16KO, KC and KPrC mice. A two-color line (black and blue bold) was used to differentiate between control and Prdm16KO mice. Statistical power was assessed by log-rank test for significance (left). The percentage of survival at the end of the observation period (right). **(B)** FFPE pancreatic sections from 4-m-old control, Prdm16KO, KC, and KPrC mice (*n* = 8–33) were stained with H&E or immunostained with antibodies to CK19 and subjected to IHC. Representative pictures are shown (top). Scale bars: 50 μm. Relative PanIN and PDAC surface areas, number of PanIN and PDAC lesions and CK19 intensity (bottom) are shown. Data are expressed as mean ± SEM, and statistical power was assessed by a two-tailed, Mann–Whitney test.

**Figure S3.**
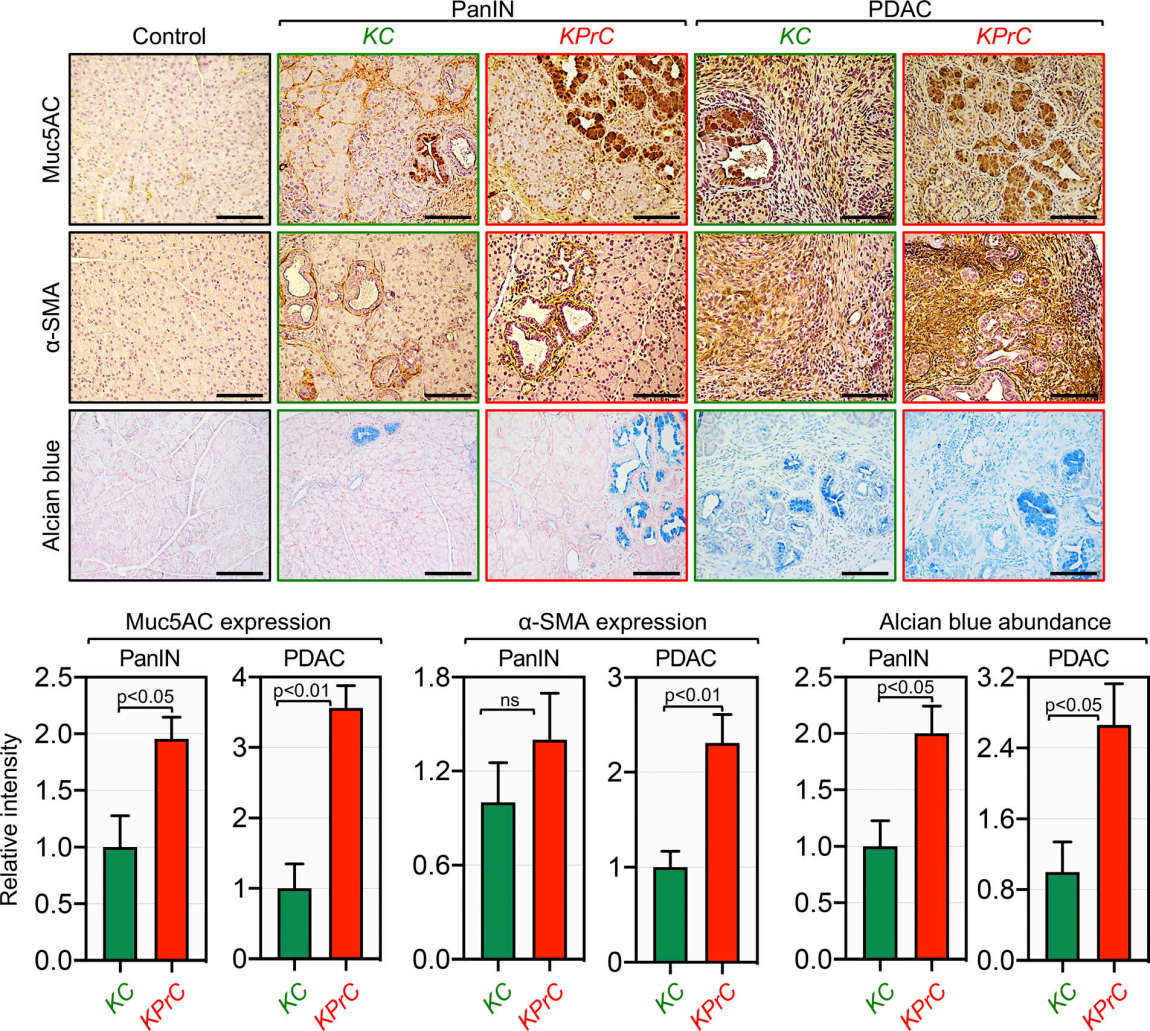
**Prdm16 ablation accelerates PDAC driven by KrasG12D.** FFPE pancreatic sections from 4-mo-old control, Prdm16KO, KC, and KPrC mice (*n* = 8–33) were stained with H&E or Alcian Blue or immunostained with antibodies to Mu5AC or α-SMA and subjected to IHC. Representative pictures are shown. Scale bars: 50 μm (top). Relative intensity of Muc5AC, α-SMA, and Alcian blue staining in areas of PanIN and PDAC lesions are shown (*n* = 13–33). Data are expressed as mean ± SEM (bottom), and statistical power was assessed by a two-tailed, unpaired Mann–Whitney test.

Next, we sought to investigate whether Prdm16 could influence PDAC progression initiated through activation of Kras signaling. The salient genetic features of PDAC originate with the near-ubiquitous gain of function mutations in KRAS in their incipient stage. However, progression to invasive PDAC in KrasG12D-bearing mice has proved to be either a protracted or unachieved process, as a small fraction of mice succumb directly to PDAC following a very long latency period (Hingorani et al., 2005; Parajuli et al., 2020; Parajuli et al., 2019; Tuveson et al., 2004). It is widely believed that the acquisition of secondary mutations in certain tumor suppressors can endow transformed cells with the growth advantage needed for disease progression. For instance, combining KrasG12D with deletion of Smad4 or TβRII has been shown to accelerate the progression of PDAC, which was thought to be conferred through disruption of TGF-β cytostatic signaling (Bardeesy et al., 2006b; Ijichi et al., 2006; Izeradjene et al., 2007). Given its role as an inhibitor of Smad signaling, we surmised that Prdm16 inactivation might suppress PDAC development and/or progression owing to the de-repression of TGF-β/Smad signaling. To probe this possibility, we generated mice harboring KrasG12D alone (KC) or in combination with conditional deletion of both alleles of Prdm16 (KPrC) and conducted comparative studies to analyze their PDAC phenotypes. Consistent with previous studies (Parajuli et al., 2020; Parajuli et al., 2019; Tuveson et al., 2004), KC mice maintained uniformly good health until around the age of 20 wk, and thereafter a fraction of mice became suddenly morbid and succumbed within days to an aggressive PDAC. Contrary to our prediction, combining Prdm16 deletion with KrasG12D instead resulted in a marked acceleration of PDAC. Kaplan-Meyer analysis showed a significant decrease in the median survival of KPrC mice as compared to KC mice (Fig. 3 A). During an observation period of 6 mo, 70% of KPrC mice succumbed to PDAC, whereas more than 76% of KC mice survived and remained free of invasive PDAC (Fig. 3 A). To confirm this finding, we conducted histopathological analysis with pancreatic sections from KPrC and KC mice of the same age that showed either relatively healthy appearance or signs of morbidity characteristic of invasive PDAC at the time of necropsy. At early stages of tumorigenesis, KPrC mice displayed a significant increase in PanIN lesions compared to KC mice, as assessed by H&E and IHC using anti-CK19 antibody (Fig. 3 B). A similar conclusion could be drawn while analyzing another ductal marker, MUC5AC, either by IHC or Alcian blue staining (Fig. S3). KPrC and KC mice with invasive PDAC also showed clear difference in both tumor architecture and reactivity to the anti-CK19 and anti-Mu5AC antibodies as well as to Alcian blue (Fig. 3 B and Fig. S3). Moreover, IHC analysis using anti-α-SMA antibody showed more extensive stroma both within and outside PDAC lesions in KPrC mice relative to KC mice (Fig. S3). An automatic-guided quantification confirmed the increase in the surface areas of PanIN and PDAC lesions in KPrC mice as compared to KC mice (Fig. 3 B). Thus, Prdm16 inactivation appeared to accelerate PDAC once it has been initiated through activation of oncogenic KrasG12D signaling.

### Requirement of Prdm16 for IPMN-to-PDAC progression

**Figure 4.**
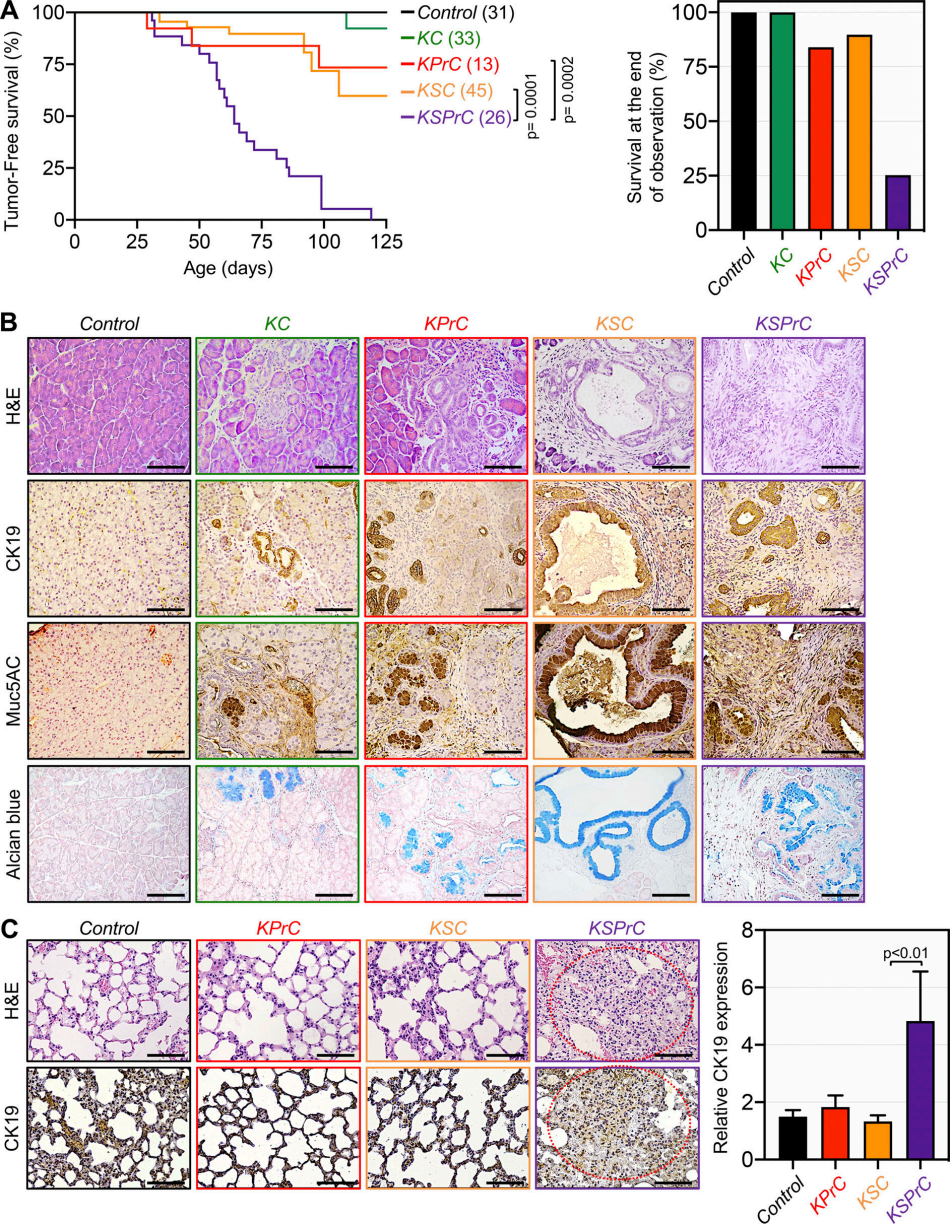
**Concomitant inactivation of Prdm16 and Smad4 shifts the progression trajectory of PDAC. (A)** Kaplan-Meier survival analysis of control, KC, KPrC, KSC, and KSPrC mice (*n* = 13–45). Statistical power was assessed by a log-rank test for significance (left). The percentage of survival at the end of the observation period (right). **(B)** FFPE pancreatic sections from 4-m-old control, KC, KPrC, KSC, and KSPrC mice (*n* = 13–45) were stained with H&E or Alcian blue or immunostained with antibodies to CK19 or Muc5AC and subjected to IHC. Representative pictures are shown. Scale bars: 50 μm. **(C)** FFPE lung sections from 4-mo-old control, KPrC, KSC and KSPrC mice (*n* = 13–45) were stained with H&E or immunostained with anti-CK19 antibody. Metastatic lesions are highlighted by blue dot-circles. Representative pictures are shown (left). Scale bars: 50 μm. Relative CK19 intensity from lung sections are shown (right). Data are expressed as mean ± SEM, and statistical power was assessed by a two-tailed Mann–Whitney test.

**Figure S4.**
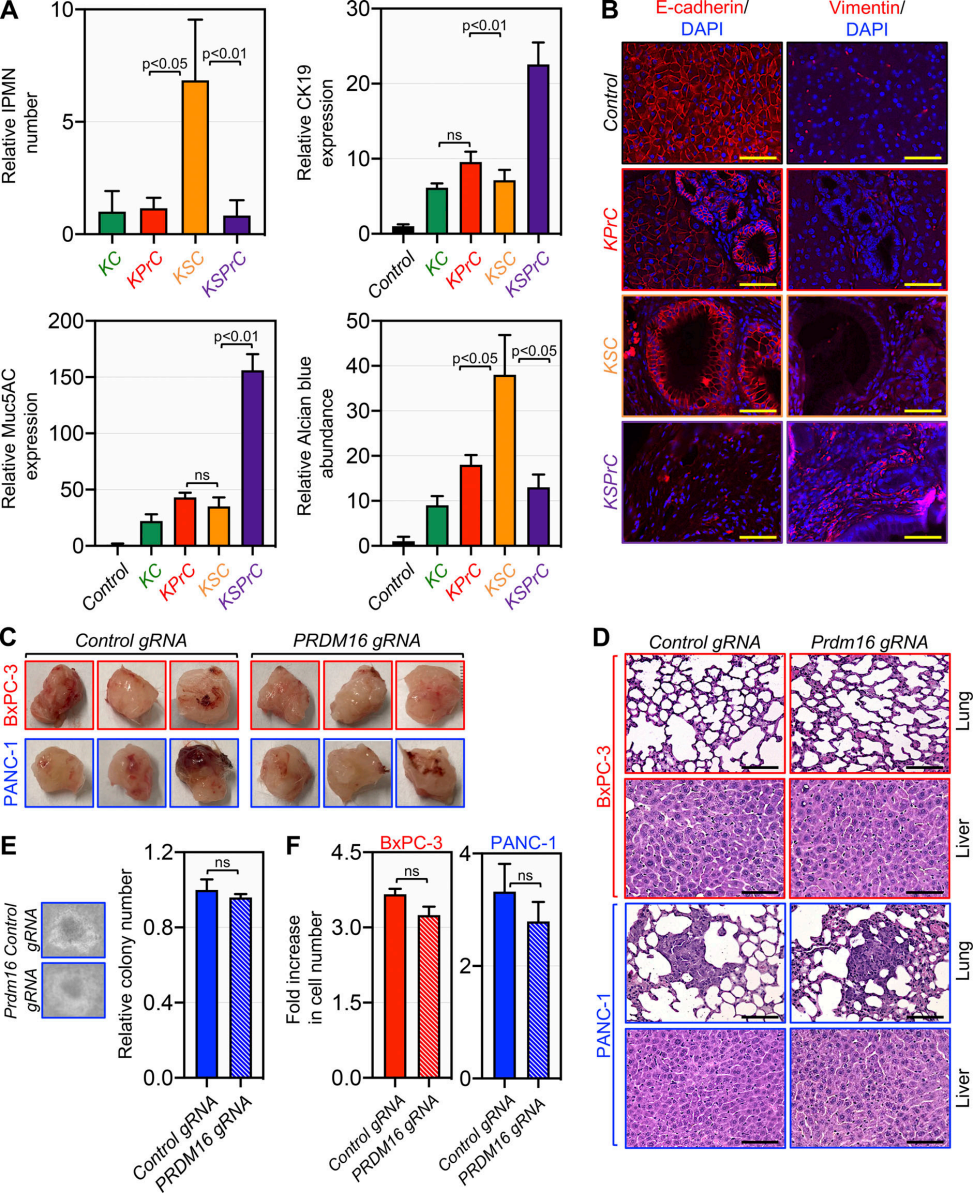
**Prdm16 inactivation in KSC mice resulted in acceleration of PDAC. (A)** FFPE pancreatic sections from 4-mo-old control, KC, KPrC, KSC, and KSPrC mice (*n* = 13–45) were stained with H&E or Alcian blue or immunostained with antibodies to CK19 or Muc5AC and subjected to IHC. Relative abundance of IPMN lesions (top left) or intensity of CK19 (top right), Muc5AC (bottom left), or Alcian blue (bottom right) are shown. Data are expressed as mean ± SEM, and statistical power was assessed by a two-tailed, unpaired Mann–Whitney test. **(B)** FFPE pancreatic sections from control, KPrC, KSC, and KSPrC mice (*n* = 13–45) were subjected to IF using antibodies to E-cadherin or vimentin. Representative pictures are shown. Scale bars: 50 μm. **(C)** Pictures of tumors harvested from NSG mice injected with isogenic PANC-1 and BxPC-3 cell lines stably expressing control or PRDM16 gRNA (*n* = 3). **(D)** FFPE liver and lung sections from NSG mice injected with isogenic PANC-1 and BxPC-3 cell lines stably expressing control or PRDM16 gRNA were stained with H&E (*n* = 3). Representative pictures are shown. Scale bars: 50 μm. **(E)** Representative pictures of soft-agar colonies formed by isogenic PANC-1 cell lines stably expressing control or PRDM16 gRNA. BxPC-3 stably expressing control or PRDM16 gRNA did not form colonies. **(F)** Cell proliferation assay of isogenic PANC-1 (day 3) and BxPC-3 (day 6) cell lines stably expressing control or PRDM16 gRNA. The fold increase in cell number at the end of the experiment relative to the seeding density is shown (*n* = 3). Data are expressed as mean ± SEM and statistical power was assessed by a two-tailed, unpaired Mann–Whitney test.

Given the inverse association between Smad4 and Prdm16 that we noticed earlier during PDAC progression (Fig. 1, E and F), we sought to extend our genetic approaches to explore whether Prdm16 could play a role, if any, in PDAC that depends on its function in TGF-β/Smad signaling. Accordingly, we generated mice with the combined deletion of Prdm16 and Smad4 in a KrasG12D background (KSPrC). KPrC, KSC, KC, and wild-type mice were used as controls. KSPrC mice were born with Mendelian frequencies, and no phenotypic differences between KSPrC and KSC mice were observed. Strikingly, however, the vast majority of KSPrC mice became stunted and morbid in appearance within 2 to 3 wk of weaning, and only 25% of them survived beyond 3 mo (Fig. 4 A). During this period, most of KPrC and KSC mice (84 and 90%, respectively) did not develop or succumb to PDAC. To elucidate the mechanism causing the acceleration of PDAC in KSPrC mice, we conducted histopathological analyses to study different stages of PDAC from the premalignant lesions to invasive adenocarcinomas. We found that KSC pancreas displayed predominantly macroscopic cystic lesions reminiscent of IPMN, as evidenced by the overall architecture as well as the high reactivity to the anti-Muc5AC and anti-CK19 antibodies as well as Alcian blue (Fig. 4 B and Fig. S4 A). In contrast, KSPrC pancreas displayed none to very few IPMN lesions (Fig. 4 B and Fig. S4 A). At the stage of full PDAC, KSPrC tumors were poorly differentiated adenocarcinomas, characterized by loss of the epithelial marker E-cadherin and acquisition of the mesenchymal marker vimentin (Fig. S3 B), which could be due either to increased accumulation of cancer associated fibroblasts or epithelial to mesenchymal transition (EMT), the latter being a general hallmark of metastasis (Pei et al., 2019). In marked contrast, KSC tumors were well differentiated with little or no change in E-cadherin or vimentin expression (Fig. S4 B), in line with previous studies that KSC mice are resistant to metastasis (Bardeesy et al., 2006b; Izeradjene et al., 2007; Whittle et al., 2015). As concomitant inactivation of Prdm16 appeared to shift the evolution of the IPMN-to-PDAC progression sequence toward the PanIN-to-PDAC progression sequence, it is tempting to speculate that Prdm16 might function at the stage of early preneoplastic lesions to influence PDAC development and progression. In support of this notion, deleting PRDM16 in two fully-transformed human PDAC cell lines (i.e., PANC-1, sufficient for SMAD4 and BxPC-3, deficient for SMAD4) did not affect their proliferative or invasive behaviors, as gauged by a combination of in vivo and in vitro assays (Fig. S4, C–F).

The poor prognosis for human PDAC is mainly due to quasi-inevitable metastasis affecting the liver and lung at the time of diagnosis (Connor and Gallinger, 2021; Hidalgo, 2010). Due to the severity of PDAC in KSPrC mice, we wondered whether concomitant deletion of Prdm16 could confer metastatic ability to the otherwise non-metastasizing PDAC tumors that typically develop in KSC mice (Bardeesy et al., 2006b; Izeradjene et al., 2007; Whittle et al., 2015). Indeed, we consistently observed the presence of metastatic lesions in the lung in all KSPrC mice that developed invasive tumors but survived until necropsy (Fig. 4 C). In contrast, no metastatic lesions were detected in KSC mice even with terminal PDAC (Fig. 4 C), as previously described (Bardeesy et al., 2006b; Izeradjene et al., 2007; Whittle et al., 2015). Confirmation of these results was obtained by IHC using an antibody to the PDAC marker CK19 (Fig. 4 C). Collectively, these data demonstrate that concomitant inactivation of Prdm16 was sufficient to confer metastatic properties on non-metastatic KSC tumors, a phenomenon that is associated with a shift from the IPMN-to-PDAC phenotype to the PanIN-to-PDAC phenotype.

### Repression of Prdm16 expression by Smad4

**Figure 5.**
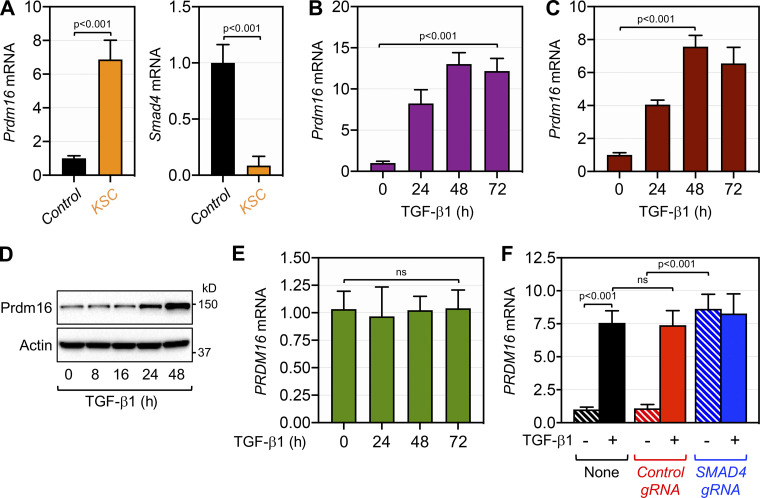
**Smad4 represses Prdm16 expression. (A)** Expression of Prdm16 (left) or Smad4 (right) in pancreas from 4-mo-old control and KSC mice (*n* = 6) was analyzed by qRT-PCR. **(B)** Expression of Prdm16 mRNA in KPC1 cells cultured in the presence or absence of TGF-β1 for various times was analyzed by qRT-PCR (*n* = 6). **(C)** Expression of PRDM16 mRNA in PANC-1 cells treated with TGF-β1 for various times was analyzed by qRT-PCR (*n* = 6). **(D)** Expression of Prdm16 protein in PANC-1 cells treated with TGF-β1 for various times was analyzed by immunoblotting. **(E)** Expression of PRDM16 mRNA in MIA-PaCa-2 cells cultured in the presence or absence of TGF-β1 for various times was analyzed by qRT-PCR (*n* = 6). **(F)** Expression of PRDM16 mRNA in isogenic PANC-1 cell lines stably transduced with control or SMAD4 gRNA lentiviruses and cultured in the presence or absence of TGF-β1 was analyzed by qRT-PCR (*n* = 6). Data in A–C, E, and F are expressed as mean ± SEM, and statistical power was assessed by a two-tailed, unpaired *t* test. Source data are available for this figure: SourceData F5.

**Figure 6.**
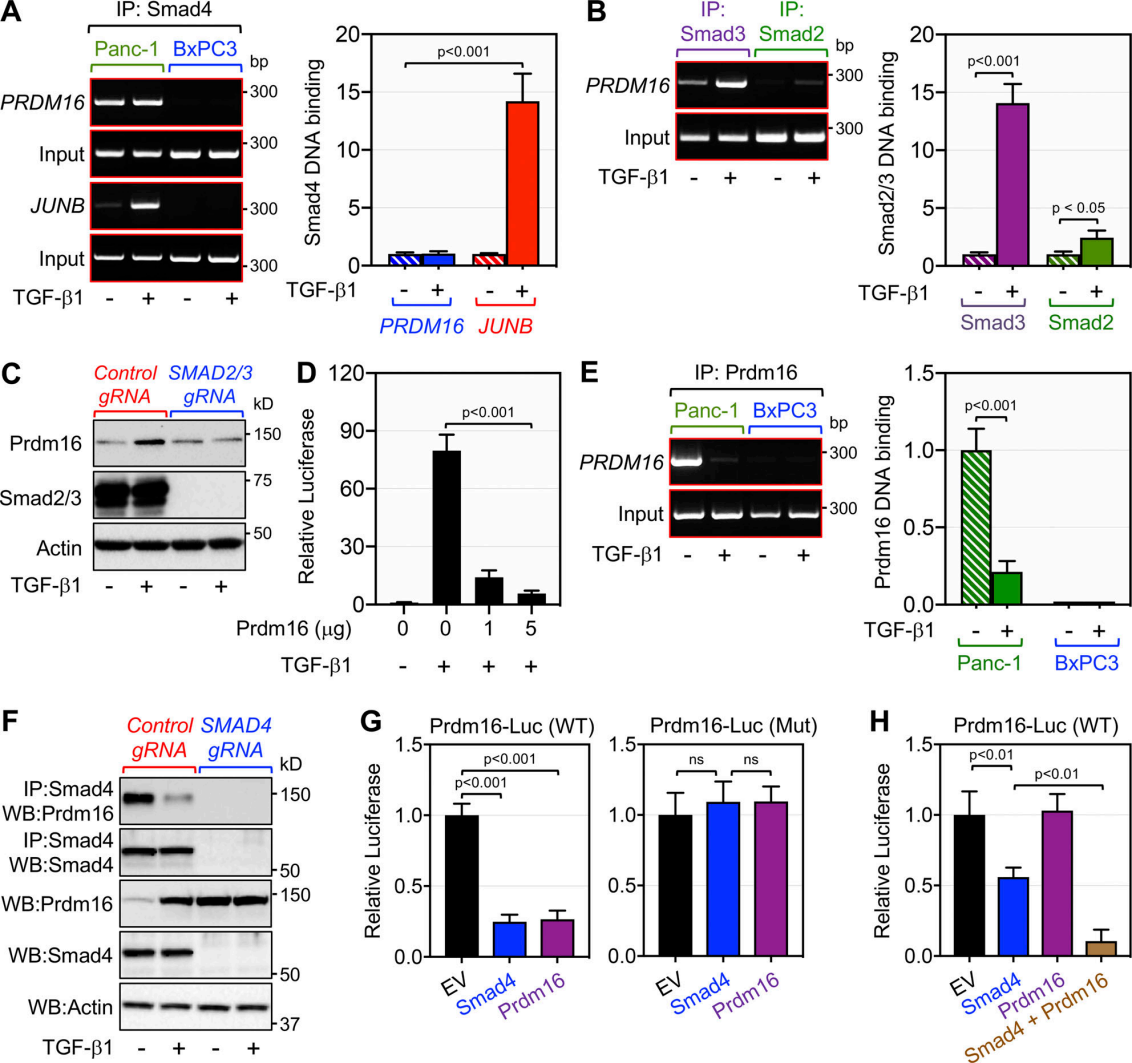
**Smad4 interacts with Prdm16 on the PRDM16 promoter to repress its own expression. (A)** Pancreatic chromatin from PANC-1 or BxPC-3 cells (*n* = 6) cultured in the presence or absence of TGF-β1 was analyzed for the binding of Smad4 to the PRDM16 or JUNB promoter by ChIP and agarose gel (left) and qPCR (right). **(B)** Pancreatic chromatin from PANC-1 cells (*n* = 6) cultured in the presence or absence of TGF-β1 was analyzed for the binding of Smad2 and Smad3 to the PRDM16 promoter by ChIP and agarose gel (left) and qPCR (right). **(C)** PANC-1 expressing control or SMAD2/3 gRNAs were cultured in the presence or absence of TGF-β1 for 48 h and analyzed for the expression of Prdm16 and Smad2/Smad3 by direct immunoblotting. **(D)** PANC-1 cells were transfected with the CAGA9-Lux gene reporter and increasing amounts of pcDNA3.1-Prdm16. 24 h after transfection, cells were treated with TGF-β1 for 16 h and then assessed for luciferase activity and normalized. **(E)** Pancreatic chromatin from PANC-1 or BxPC-3 cells (*n* = 6) cultured in the presence or absence of TGF-β1 was analyzed for the binding of Prdm16 to the PRDM16 promoter by ChIP and agarose gel (left) and qPCR (right). **(F)** PANC-1 expressing control or SMAD4 gRNA were treated with TGF-β1 for 48 h and then analyzed for the interaction of Prdm16 with Smad4 by co-immunoprecipitation (IP) followed by immunoblotting (WB). Expression of Prdm16 was also analyzed by direct immunoblotting. **(G)** PANC-1 cells were transfected with the wild-type (left) or mutated Prdm16-Lux (right) reporter together with empty vector, pcDNA3.1-Prdm16 or pCMV5-HA-Smad4. 48 h after transfection, cells were assessed for luciferase activity and normalized (*n* = 6). **(H)** BxPC-3 cells were transfected with the wild-type Prdm16-Lux reporter together with the indicated combinations of empty vector (EV), pcDNA3.1-Prdm16, and pCMV5-HA-Smad4. 48 h after transfection, cells were assessed for luciferase activity and normalized (*n* = 6). Data in A, B, D, E, G, and H are expressed as mean ± SEM, and statistical power was assessed by a two-tailed, unpaired *t* test. Source data are available for this figure: SourceData F6.

To investigate the molecular mechanisms by which Prdm16 controls PDAC progression and metastasis in the context of a Smad4 null background, we took advantage of our earlier IHC analysis showing that Smad4 deficiency in KSC mice was associated with a persistent de-repression of Prdm16 during the progression from IPMN to PDAC (Fig. 1 E). We surmised that Smad4 might function either directly or indirectly to repress Prdm16 expression, which in turn impacts the progression trajectory of PDAC. We initially conducted qRT-PCR experiments using KSC mice, and found that the increase in Prdm16 expression was mediated at least via gene expression (Fig. 5 A). Because Smad4 functions as an essential component of TGF-β signaling (David and Massagué, 2018; Feng and Derynck, 2005; Massagué, 2008), we next wondered whether activation of TGF-β signaling could repress Prdm16 expression, as does Smad4. To our surprise, treating mouse PDAC cells KPC1 or human PDAC cells PANC-1 with TGF-β1 instead elicited a marked increase in Prdm16 expression (Fig. 5, B–D). As a specificity control, TGF-β1 treatment failed to induce Prdm16 expression in the human PDAC cell line MIA-PaCa-2 (Fig. 5 E), which lacks a functional TGF-β receptor (Freeman et al., 1995). To determine whether the effect of TGF-β1 is mediated via Smad4, we conducted comparative experiments using PANC-1 cells deleted of SMAD4 by CRISPR/CAS9. We found that ablating SMAD4 resulted in a marked increase in the steady-state expression of Prdm16 mRNA and protein (Fig. 5 F, see also Fig. 6 F), confirming the ability of endogenous Smad4 to repress PRDM16 expression in human cells. Intriguingly, challenging cells with TGF-β1 did not further increase Prdm16 expression in cells deleted of SMAD4 as compared to cells expressing the control gRNA (Fig. 5 F; see also Fig. 6 F), implying that SMAD4 inactivation is sufficient to mimic the effects of TGF-β1 stimulation.

**Figure 7.**
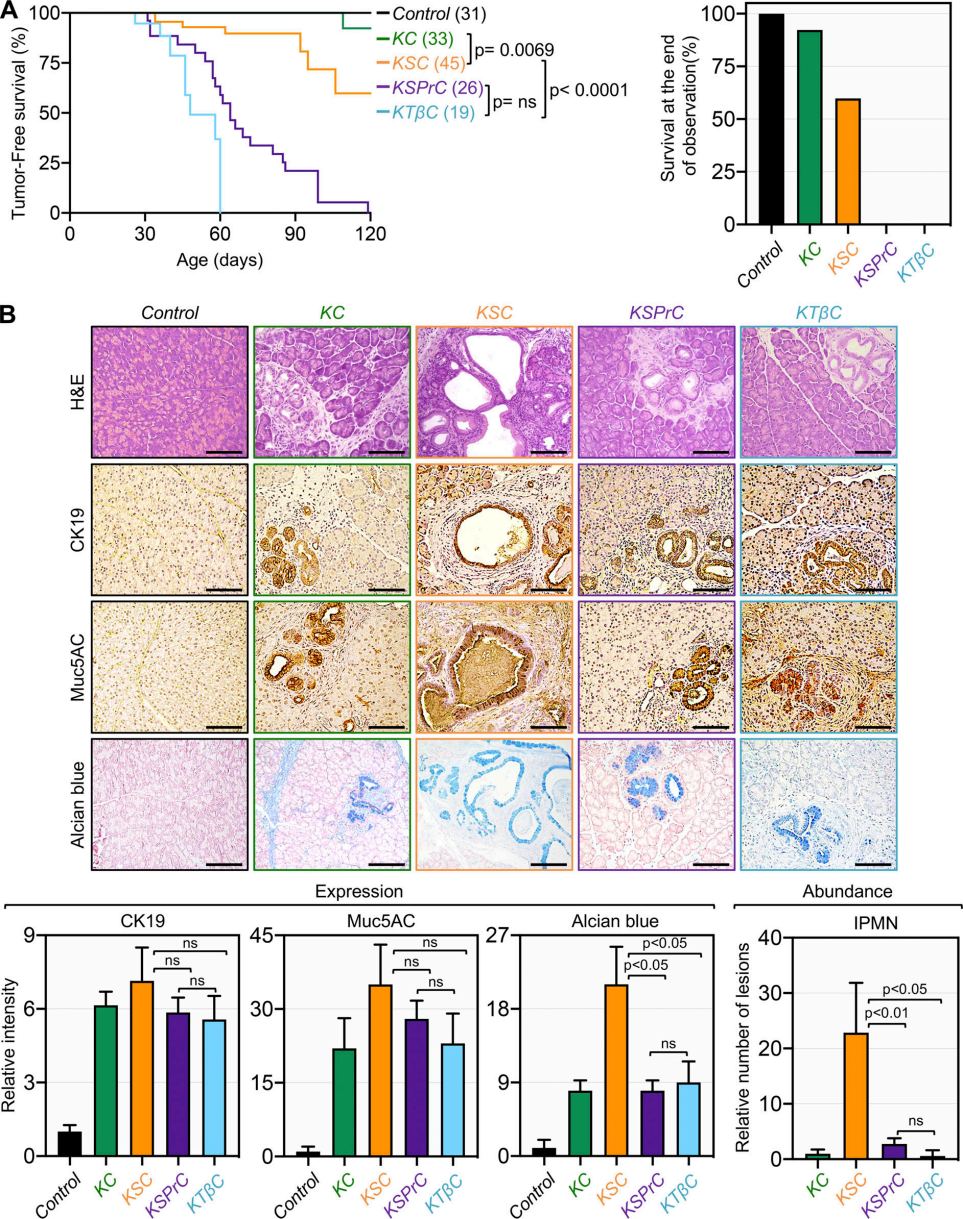
**Concomitant ablation of Prdm16 and Smad4 recapitulates the global inactivation of TGF-β signaling through ablation of TβRII. (A)** Kaplan-Meier survival analysis of control, KC, KSC, KSPrC, and KTβC mice (*n* = 19–45). Statistical power was assessed by a log-rank test for significance (left). The percentage of survival at the end of the observation period (right). **(B)** FFPE pancreatic sections from 1- to 4-mo-old control, KC, KSC, KSPrC, and KTβC mice (*n* = 19–45) were stained with H&E or Alcian blue, or immunostained with antibodies to CK19 or Muc5AC and subjected to IHC. Scale bars: 50 μm (top). Relative intensity of CK19, Muc5AC, and Alcian Blue or relative IPMN abundance are shown (bottom). Data are expressed as mean ± SEM, and statistical power was assessed by a two-tailed, unpaired Mann–Whitney test.

Previous studies have shown that Smad proteins can stimulate or repress expression of TGF-β responsive genes through direct binding to their promoter (David and Massagué, 2018; Feng and Derynck, 2005; Massagué, 2008). In addition, a substantial fraction of Smad4 has been shown to localize in the nucleus in the absence TGF-β stimulation, but the physiopathological significance of this phenomenon remains unknown (Pierreux et al., 2000). Because SMAD4 ablation in PANC-1 cells was sufficient to recapitulate the stimulatory effects of TGF-β signaling on PRDM16 expression, we initially reasoned that Smad4 might bind to and repress the PRDM16 promoter at steady state, and that TGF-β signaling activation might displace Smad4 from the PRDM16 promoter. Accordingly, we conducted ChIP experiments, focusing on Smad conserved binding elements (SBE) within the PRDM16 promoter that we identified through an in-silico analysis. Using chromatin from PANC-1 cells, we detected a strong binding of Smad4 to the PRDM16 promoter at steady state (Fig. 6 A). This binding is specific, as there was no signal in the human PDAC cell line BxPC-3 (Fig. 6 A), which bears natural homozygous deletion of SMAD4 (Duda et al., 2003). Interestingly, treating PANC-1 cells with TGF-β1 had little or no effect on the binding of Smad4 to the PRDM16 promoter despite eliciting a strong activation of this pathway, as assessed by the increased binding of Smad4 to the promoter of JUNB (Fig. 6 A), a well-characterized TGF-β target gene (Sundqvist et al., 2018). This observation, together with our gene expression experiments, strongly suggests that TGF-β signaling might involve other players that act in partnership with Smad4 to repress Prdm16 expression. To explore this possibility, we conducted ChIP experiments using antibodies to Smad2 and Smad3, as both transcription factors are known to interact with Smad4 in response to TGF-β signaling (David and Massagué, 2018; Feng and Derynck, 2005; Massagué, 2008; Massagué et al., 2005). We detected a slight but significant increase in the binding to Smad2 to the PRDM16 promoter in PANC-1 cells upon stimulation with TGF-β1 (Fig. 7 B). In contrast, TGF-β1 stimulation induced a massive increase in the binding of Smad3 to the PRDM16 promoter (Fig. 6 B). Concomitant deletion of SMAD2 and SMAD3 in PANC-1 cells resulted in almost complete blockade in TGF-β-induced Prdm16 expression (Fig. 6 C). Although this finding provides a potential mechanism by which TGF-β signaling could induce Prdm16 expression, it failed to explain why deletion of Smad4 in KSC mice leads to the derepression of Prdm16. Based on the literature (Chuikov et al., 2010; Stine et al., 2019; Takahata et al., 2009) and our data that Prdm16 can repress Smad transcriptional activity in human PANC-1 cells (Fig. 6 D), we considered the possibility that Smad4 might recruit Prdm16 to its own promoter, thereby leading to Prdm16 repression. Indeed, we detected a strong binding of Prdm16 to its promoter in PANC-1 cells at steady state, and this was almost completely suppressed upon TGF-β1 stimulation (Fig. 6 E), strongly suggesting that TGF-β signaling activation might dislodge Prdm16 from its promoter. In comparison, we were not able to detect any binding of Prdm16 to its promoter in BxPC-3 cells (Fig. 6 E), attesting to the specificity of our experiments, and further providing strong evidence supporting the notion that Smad4 functions to recruit Prdm16 to its promoter to repress its expression. To corroborate these findings, we conducted co-immunoprecipitation assays using PANC-1 cells, and detected a strong interaction between Prdm16 and Smad4, which was inhibited upon treatment of cells with TGF-β1 (Fig. 6 F). Such interaction was not detected in PANC-1 cells deleted of SMAD4 (Fig. 6 F), attesting to the specificity of the approach. Finally, we generated a reporter construct in which luciferase expression is under the control of either wild-type or mutated (SBE) PRDM16 promoter (Prdm16-Lux). We found that Smad4 was able to repress expression from the wild-type PRDM16 promoter in PANC-1 cells (Fig. 6 G). More importantly, expression of Prdm16 was also able to suppress luciferase expression from the wild-type PRDM16 promoter, and this effect was completely lost when the SBE mutated promoter was used in the assay (Fig. 6 G). Finally, expression of Prdm16 was able to repress the wild-type PRDM16 promoter in BxPC-3 cells only when Smad4 was co-expressed (Fig. 6 H). Overall, these findings revealed that Smad4 functions as a potent repressor of Prdm16, therefore providing a mechanistic explanation as to why KSC mice display high expression of Prdm16.

### Concomitant inactivation of Prdm16 and Smad4 recapitulates the global inactivation of TGF-β signaling

Both SMAD4 and TβRII are frequently inactivated in human PDAC, and landmark genetic experiments have shown that inactivation of either Smad4 or TβRII accelerates KrasG12D-driven PDAC (Bardeesy et al., 2006b; Ijichi et al., 2006; Izeradjene et al., 2007). To date, it remains largely unknown whether inactivation of Smad4 or TβRII could differentially impact the dynamics or trajectory of PDAC progression. Our mechanistic data that inactivation of Smad4 recapitulates the effects of TGF-β signaling on Prdm16 expression provided us with a unique platform to address this issue. To do so, we conducted an in-depth comparative analysis of the PDAC phenotypes in mice with homozygous deletion of TβRII (KTβC), KSC, and KSPrC mice side-by-side. KC and wild-type mice were used as controls. Kaplan-Meier analysis showed that KTβC mice developed lethal PDAC much more earlier than KSC mice, often succumbing to the disease within 4 wk of age and none survived beyond 17 wk, whereas 60% of KSC mice survived within this observation period (Fig. 7 A). This observation indicates that global inactivation of TGF-β signaling through TβRII ablation is more efficient at deepening PDAC progression than inactivation of canonical TGF-β/Smad signaling through Smad4 ablation. More importantly, we found that KSPrC mice succumbed to lethal PDAC with kinetics approaching that of KTβC mice (Fig. 7 A), suggesting that simultaneous inactivation of Smad4 and Prdm16 might be sufficient to recapitulate the global inactivation of TGF-β signaling.

**Figure S5.**
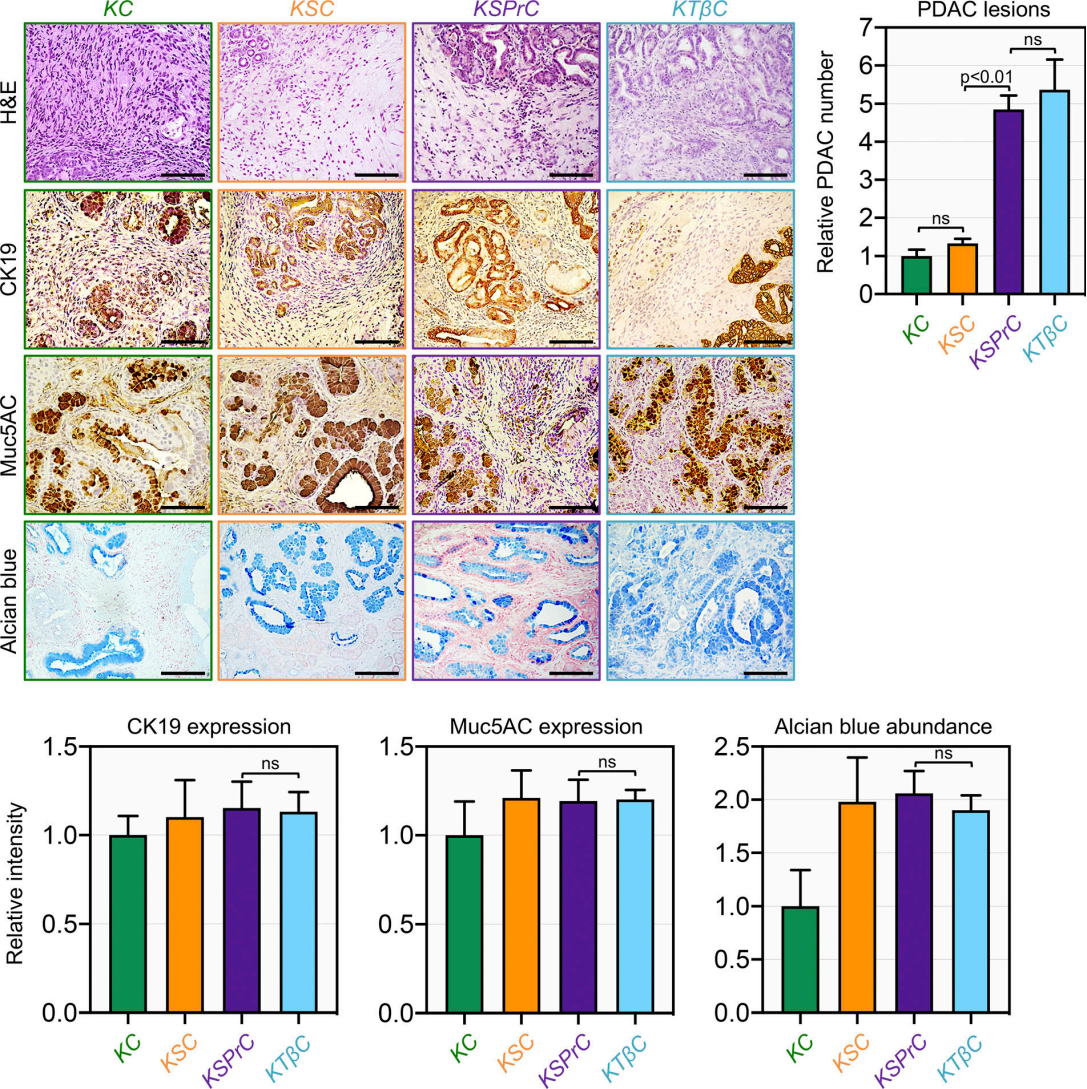
**Alterations in TGF-β signaling result in different PDAC trajectories.** FFPE pancreatic sections from 1–4-mo-old KC, KSC, KSPrC, and KTβC mice (*n* = 19–45) with full-blown PDAC were stained with H&E or Alcian blue, or immunostained with antibodies to CK19 or Muc5AC and subjected to IHC. Representative pictures are shown. Scale bars: 50 μm (top left). Relative number of PDAC lesions (top right). Relative intensity of CK19, Muc5AC, and Alcian blue staining in PDAC lesions (bottom). Data are expressed as mean ± SEM, and statistical power was assessed by a two-tailed, unpaired Mann–Whitney test.

Next, in light of our earlier findings that concomitant inactivation of Prdm16 was able to shift the evolution of the IPMN-to-PDAC phenotype in KSC mice toward the PanIN-to-PDAC phenotype, we wondered whether ablation of TβRII or Smad4 could differentially affect the nature of the premalignant lesions leading to PDAC, and if so, whether this event depends on Prdm16. Thus, we conducted histopathological analyses to compare the PDAC phenotypes in KSPrC, KTβC and KSC mice both at the levels of pre-malignant and full-blown PDAC lesions. H&E staining showed that KTβC tumors displayed uniformly poorly differentiated architecture, which is consistent with the rapid development of invasive PDAC in these mice (Fig. S5). Nevertheless, using KTβC mice before displaying signs of invasive PDAC, we consistently noticed the presence of premalignant lesions that display the classical features of PanINs, as gauged by IHC using antibodies to CK19 and Muc5AC (Fig. 7 B). Interestingly, KSPrC mice displayed similar cancerous phenotype as KTβC mice, both in terms of PanIN and PDAC lesions (Fig. 7 B and Fig. S5). In contrast, KSC mice consistently showed abundant and large IPMN lesions that exhibit high reactivity to the anti-Muc5AC antibody and Alcian blue (Fig. 7 B), which is in agreement with previous studies that KSC mice develop IPMN premalignant lesions rather than PanIN lesions (Bardeesy et al., 2006b; Izeradjene et al., 2007; Whittle et al., 2015). Taken together, these findings strongly suggest that inactivation of the entire TGF-β/Smad pathway promotes PanIN-to-PDAC progression, whereas inactivation of Smad4 promotes IPMN-to-PDAC progression. In addition, since concomitant ablation of Prdm16 and Smad4 resulted in highly aggressive PDAC similar to what was observed in KTβC mice, we suggested that global inactivation of TGF-β signaling might simultaneously inactivate both Smad4 and Prdm16.

## Discussion

**Figure 8.**
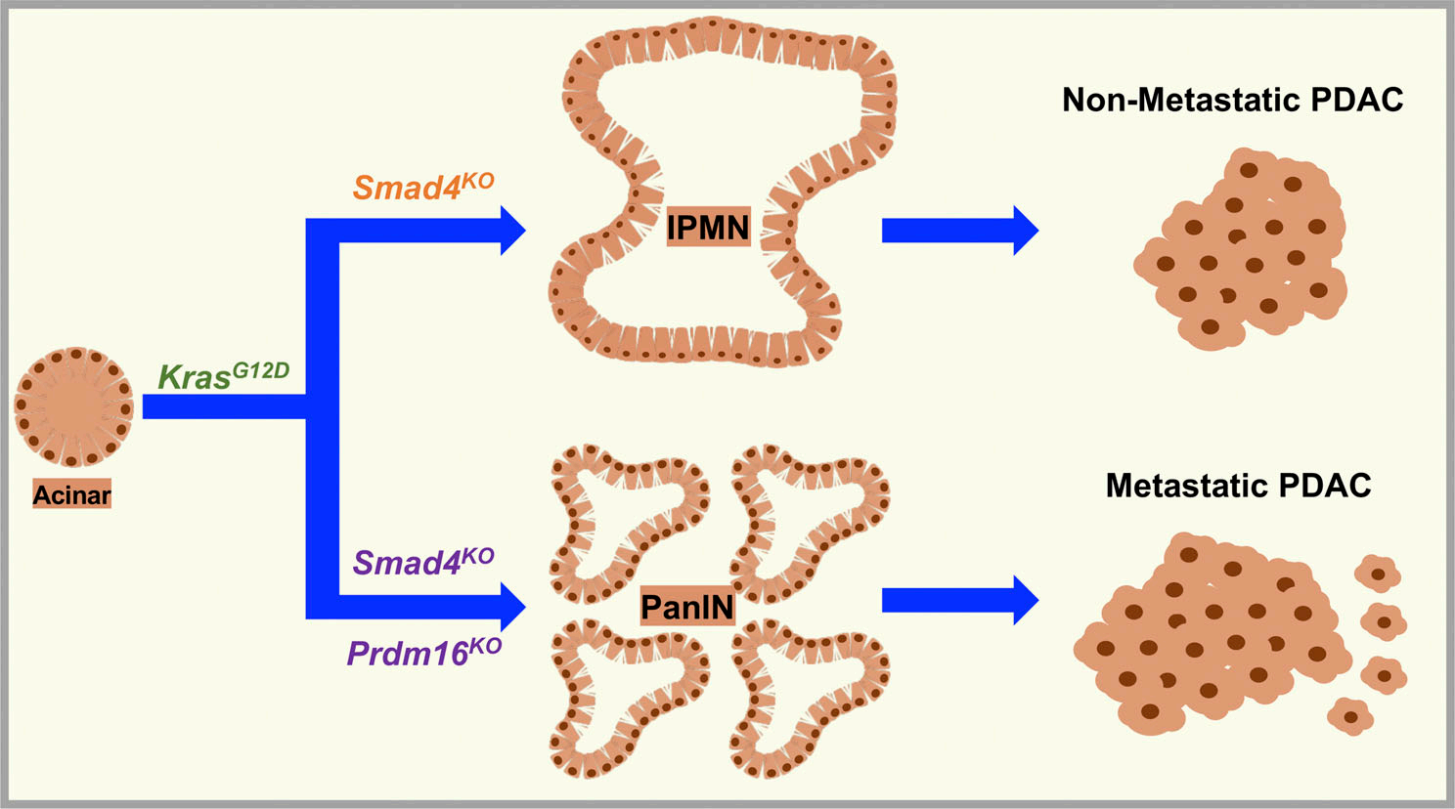
Model for the functional interaction between Smad4 and Prdm16 during PDAC formation and progression.

Prdm16 belongs to the PR domain-containing protein family of transcription factors, which control a plethora of essential cellular processes, including specification of cell lineage during development (Chi and Cohen, 2016). Prdm16 was first identified in leukemia, where truncation mutants lacking functional domains behaved as oncogenic (Zhou et al., 2016), providing the first indication that Prdm16 might function as a tumor suppressor. In addition its involvement in leukemia, several studies have subsequently shown that Prdm16 controls brown fat cell differentiation as well as dedifferentiation of white fat to beige fat (Harms et al., 2015; Hiraike et al., 2017; Seale et al., 2008; Seale et al., 2007). Moreover, Prdm16 is required for stemness in multiple tissues, including hematopoietic and nervous systems (Chi and Cohen, 2016). Germline deletion of Prdm16 in mice impairs the maintenance of neural and hematopoietic stem cells during fetal development, resulting in neonatal death (Shimada et al., 2017). As such, this lethal phenotype hampered any further investigation to delineate a possible role of Prdm16 in cell fate determination in other organ systems, such as pancreas, where the same progenitor cells give raise to all pancreas lineages, e.g., ductal, acinar, and islet (Gu et al., 2003). In this study, we found that conditional deletion of Prdm16 in early pancreatic progenitor cells had no discernible impact on animal health or pancreas physiology, indicating that Prdm16 is dispensable for pancreas development and function. Because mutational inactivation of PRDM16 has been shown to be associated with leukemia (Zhou et al., 2016), we went on to explore whether Prdm16 could contribute to the pathogenesis and/or progression of PDAC, in which acquisition of oncogenic KRAS endows acinar cells with stemness traits that facilitate their differentiation toward a ductal-like lineage, thereby culminating in acinar-to-ductal metaplasia and attendant emergence of premalignant lesions (Bardeesy et al., 2006a; Gu et al., 2003; Park et al., 2008; Tuveson et al., 2004). Progression of premalignant lesions either follows the PanIN-to-PDAC sequence, MCN-to-PDAC, or IPMN-to-PDAC sequence, depending on the nature of the secondary genetic events (Bardeesy et al., 2006a; Bardeesy et al., 2006b; Gu et al., 2003; Tuveson et al., 2004). Yet, among the most studied secondary genetic alterations in PDAC, only Smad4 inactivation stood out as the main mechanism that enables progression through the IPMN-to-PDAC sequence (Bardeesy et al., 2006b; Whittle et al., 2015). To the best of our knowledge, how Smad4 inactivation facilitates this IPMN-to-PDAC transition phenotype has never been addressed experimentally. Using the KrasG12D-based mouse model of PDAC, we confirmed that KSC mice develop mostly IPMN lesions as described initially (Bardeesy et al., 2006b) rather than MCN lesions described in a subsequent study (Izeradjene et al., 2007). Most importantly, we found that concomitant ablation of Prdm16 and Smad4 (KSPrC) resulted in highly aggressive tumors, which develop with very short latencies to the full-blown PDAC and frequently metastasize to the lung, a site associated with the human disease (Connor and Gallinger, 2021; Hidalgo, 2010). Comprehensive histopathological analyses revealed that these tumors follow the PanIN-to-PDAC progression route rather than the IPMN-to-PDAC progression route that proceeds with ablation of Smad4 alone. Because inactivating Smad4 led to the increased expression of Prdm16, we proposed a model in which Prdm16 functions as a molecular switch to dictate whether the malignant transformation process follows the IPMN-to-PDAC route or the PanIN-to-PDAC route (Fig. 8). This model also posits that Prdm16 might function to suppress PDAC pathogenesis at very early stages of the malignancy. In further support of this notion, we found that ablating PRDM16 in the human PDAC cancer cell lines BxPC-3 and PANC-1 did not influence their proliferative or metastatic behaviors, as evidenced using a variety of in vivo and in vitro tumor growth and invasion assays. In light of these findings, a more comprehensive investigation using genetic and histological approaches are needed to firmly establish whether Prdm16 indeed elicits its tumor suppressor activity at early stages, and if so, whether this occurs through direct effects on cancer cell growth or tumor microenvironment reprogramming. As such, our findings open up unique frameworks that would ultimately leverage general efforts to unravel mechanistic paradigms of PDAC, for which very limited therapeutic interventions are currently available.

Accumulating evidence suggests that Prdm16 functions as a potent inhibitor of TGF-β/Smad signaling under various physiological contexts (Chuikov et al., 2010; Stine et al., 2019; Takahata et al., 2009). TGF-β/Smad signaling is well known to play a dual role during cancer progression, functioning at early stages as a tumor suppressor to restrict the malignant transformation, and at late stages as a tumor promoter to facilitate cell invasion and metastasis (Feng and Derynck, 2005). To date, the most appealing speculations as to TGF-β dual function during PDAC progression have been that loss of the TGF-β cytostatic function enables cells to escape growth-inhibitory regulation, which would ultimately culminate in malignant transformation (David et al., 2016; Feng and Derynck, 2005; Massagué, 2008). Once the tumor has developed, other TGF-β responses unrelated to its cytostatic function then supposedly prevail presumably in a manner that facilitates PDAC invasion and metastasis (Bardeesy et al., 2006b; Feng and Derynck, 2005; Ijichi et al., 2006; Massagué, 2008). Interestingly, high levels of TGF-β expression in human PDAC strongly correlates with poor prognosis (Friess et al., 1993; Parajuli et al., 2019), which raises a conundrum as to whether activation of TGF-β signaling could contribute directly to malignant transformation in addition to driving cell invasion and metastasis. However, subsequent studies have shown that Smad4 inactivation in the context of KrasG12D (KSC) led to the acceleration of PDAC (Bardeesy et al., 2006b; Izeradjene et al., 2007), unequivocally confirming the tumor suppressor role of TGF-β signaling in PDAC. Nevertheless, the tumors deficient for Smad4 retained epithelial differentiation and manifested an attenuated metastatic potential (Bardeesy et al., 2006b; Whittle et al., 2015), which is also in favor of a tumor promoter role of TGF-β signaling. So far, definitive experimental evidence on whether inactivation of canonical TGF-β/Smad signaling per se is sufficient to suppress PDAC invasion and metastasis in an irreversible manner is still lacking. Here, we found that ablating Prdm16 in a Smad4 null-background was sufficient to render the PDAC tumors again highly invasive and metastatic. Intriguingly, concomitant ablation of Prdm16 in KSC mice also resulted in a shift from IPMN to PanIN, which could conceivably contribute to metastasis in KSPrC mice, as the vast majority of PDAC GEMMs that develop PanINs also develop highly metastatic PDAC, including KSPC mice (Smad4 deletion and p53.R172H expression), which behave similarly to our KSPrC mice (Bardeesy et al., 2006a; Bardeesy et al., 2006b; Tuveson et al., 2004; Whittle et al., 2015). These findings, together with the observation that Prdm16 expression is lost during late stages of PDAC, highlight Prdm16 as a key player in PDAC progression and metastasis when Smad4 is inactivated. Because TGF-β signaling activation leads to the accumulation of Prdm16 through the suppression of Smad4 inhibitory effects, one would speculate that Smad4 and Prdm16 might function in the same signaling network that integrates the TGF-β tumor promoter effects during PDAC progression. However, it is also conceivable that Prdm16 might function to suppress metastasis induced by other TGF-β superfamily members, such as Activins and BMPs, which are known to signal through Smad4, and can enhance malignancy and promote cancer metastasis in a variety of human malignancies (Attisano and Wrana, 2000; Feng and Derynck, 2005; Pickup et al., 2017). As such, a comprehensive investigation of the mechanisms by which Smad4 and Prdm16 interact to influence PDAC progression may uncover the existence of additional key players and/or pathways that are amenable to therapeutic interventions.

Perhaps the most intriguing finding in this study was the persistent increase in Prdm16 expression during the progression from IPMN to PDAC in KSC mice, which at first glance seems to support a hypothesis in which Smad4 might function as a repressor of Prdm16 during PDAC progression, and hence conceivably that canonical TGF-β/Smad signaling might also repress Prdm16 expression. Quite unexpectedly, we found that activation of TGF-β signaling did not repress Prdm16 expression, but rather resulted in a strong accumulation of both Prdm16 mRNA and protein both in KSC mice and human PANC-1 cells. Noteworthy, we also detected relatively high expression in the stromal compartment, which likely occurs because of the increased TGF-β signaling, which is known to take place during PDAC progression and contribute to the desmoplastic stroma of this malignancy (Friess et al., 1993). In efforts to probe the underlying mechanisms, we found that inactivating Smad4 was sufficient to recapitulate the effects of TGF-β signaling, inducing Prdm16 expression to an extent similar to that elicited by TGF-β1. Based on these observations, we reasoned that activation of TGF-β signaling might relieve the transcriptional repression imposed by Smad4 on the Prdm16 promoter. However, although we found that Smad4 associated strongly with the PRDM16 promoter at steady state, this binding was not affected by the activation of TGF-β1 signaling, indicating that other factors are involved in TGF-β-mediated Prdm16 expression. Probing this possibility, we detected a strong binding of Prdm16 to its own promoter at steady state, which was almost completely abolished by TGF-β stimulation, suggesting that activation of TGF-β signaling might dislodge Prdm16 from its own promoter. Of note, Prdm16 failed to bind to its promoter in cells deficient for SMAD4, suggesting that Smad4 might associate with and recruit Prdm16 to the PRDM16 promoter. Because Prdm16 has been shown to function as a potent transcriptional repressor in various contexts (Pinheiro et al., 2012; Seale et al., 2008; Seale et al., 2007; Stine et al., 2019; Takahata et al., 2009), we proposed a model in which Prdm16 mediates its own repression once it has been recruited to its promoter by Smad4. While these data demonstrate for the first time that Prdm16 can repress its own expression, we cannot exclude the possibility that other mechanisms might also contribute to this phenomenon. Despite this limitation, our study sheds light on a previously uncharacterized interplay between Smad4 and Prdm16, which appears to dictate the progression trajectory of PDAC. Going forward, we anticipated that our discovery will guide forthcoming studies seeking to understand mechanistic paradigms of PDAC, which could ultimately pave the way for innovative therapeutic breakthroughs to curb this deadly disease.

## Materials and methods

### Plasmids

The CAGA9-Lux gene reporter construct was previously described (Seo et al., 2006). The expression vector pcDNA3.1-Prdm16 was a gift from Dr. Bruce Spiegelman (#15503; Addgene). The expression vector pCMV5-HA-Smad4 was a gift from Dr. Joan Massague. To generate the Prdm16-Lux reporter, genomic fragments (1,391 bp) upstream of the transcription initiation site (SST) of the PRDM16 gene (based on gene association NM_022114 and Eukaryotic Promoter Database, epd.epfl.ch) was amplified by the Genomic-GC PCR amplification kit (BD Biosciences) using human genomic DNA obtained from PANC-1 cells. Unique KpnI and XhoI sites were incorporated at the 5′ and 3′ ends of the sequence, respectively, to simplify directional cloning into KpnI and XhoI sites in the reporter plasmid, pGL3-basic (Promega). Introduction of inactivating mutation into the SBE sequence (−41 bp from SST) was generated by PCR using the QuickChange Site-Directed Mutagenesis kit according to the manufacturer’s instructions (Stratagene). The lentiCRISPRV2 expression vectors encoding SMAD4 and PRDM16 gRNAs were purchased from GenScript. The lentiCRISPRV2 expression vectors encoding SMAD2 and SMAD3 gRNAs were generated using lentiCRISPRV2 hygro (#98291; Addgene) and primers with sequences generated using the Synthego Design tool. All cloned cDNAs and their corresponding mutants were checked by sequencing.

#### Sequences of gRNAs

##### SMAD4

5′-TTC​TTC​CTA​AGG​TTG​CAC​AT-3′; 5′-AAT​ACA​CTT​ACC​AGG​ATG​AT-3′.

##### PRDM16

5′-CTC​GTA​CGG​CGA​GCC​CTC​CT-3′; 5′-AGG​GGT​CTT​ACC​GTC​CAG​GC-3′.

##### SMAD2

5′-TGG​CGG​CGT​GAA​TGG​CAA​GA-3′; 5′-TTC​ACA​ACT​GGC​GGC​GTG​AA-3′.

##### SMAD3

5′-CAC​CTG​CAA​CCG​GCC​ATC​CA-3′; 5′-ACA​CCT​GCA​ACC​GGC​CAT​CC-3′.

### Antibodies

Chromatin immunoprecipitation (ChIP), immunoblotting, immunofluorescence, or immunohistochemistry were performed using the following antibodies: anti-α-SMA (#19245T; Cell Signaling); anti-β-Actin (#64225332; Bio-Rad), anti-amylase (#ab21156; Abcam), anti-chromogranin-A (#ab45179; Abcam), anti-cytokeratin 19 (#ab52625; Abcam), anti-E-cadherin (#3195S; Cell Signaling), anti-glucagon (#2760; Cell Signaling), anti-insulin (#4590; Cell Signaling), anti-JunB (#3753; Cell Signaling), anti-Muc5AC (#ab3649; Abcam), anti-Prdm16 (#ab202344 and #ab106410; Abcam), anti-Smad2 (#5339; Cell Signaling), anti-Smad3, (#9523; Cell Signaling), anti-Smad4, (#46535; Cell Signaling), anti-Smad4 (#sc-7966; Santa Cruz), anti-Smad2/3 (#8685; Cell Signaling), and anti-vimentin (#5741S; Cell Signaling).

### Cell lines and culture

HEK293T, MIA-PaCa-2, BxPC-3, and PANC-1 cell lines were obtained from the American Type Culture Collection (ATCC). They were cultured in Dulbecco’s modified Eagle’s medium (DMEM) supplemented with 10% fetal bovine serum (#S11150; FBS, Atlanta Biologicals), antibiotics (#P4458; Gibco) and L-glutamine (#17921004; Corning). The murine pancreatic cancer cell line KPC1 was originally described in our recent publication (Parajuli et al., 2018). The cell line was established from a KP53 mouse, which harbored KrasG12D and one conditional allele of Trp53 (LSL-KrasG12D;LSL-Trp53fl/+;Pdx1-Cre). Freshly isolated specimen from the KP53 mouse with terminal PDAC was gently dissected, minced with scissors, and digested with Dispase II at 2.4 U/ml (#4942078001; Sigma-Aldrich) and Collagenase D at 0.5 mg/ml (#11088858001; Sigma-Aldrich) for 1 h at 37°C in an atmosphere of 5% CO2. Then, cells were washed three times with PBS, suspended in RPMI 1540 containing 20% FCS, and seeded on fibronectin-coated plates. Cell colonies were subsequently passaged by trypsinization, pooled, and propagated in DMEM supplemented with 10% FBS, antibiotics, and L-glutamine. To generate the PANC-1-SMAD4KO and PANC-1-SMAD2/3KO cell lines, cells were transduced with the corresponding lentiCRISPRV2-gRNA lentiviruses, selected with puromycin (for SMAD4) or hygromycin (for SMAD2/3), and all resistant clones were pooled and expanded as a single population. Lentiviruses were produced by transfecting HEK293T cells with lentiviral constructs and the One-Step Lentivirus Packaging System as described by the manufacturer (#631275; Takara). Lentiviral particles in the conditioned media were harvested after a period of 48–72 h. The conditioned media were then cleaned of cell debris by centrifugation at 5,000×*g* for 15 min, filtered through a 0.45-μm filter, and used immediately for cell transduction.

### In vitro and in vivo cell proliferation assays

For the soft agar assay, cell culture dishes (p60) were first prepared using complete DMEM media containing 0.6% agarose (#16500500; Thermo Fisher Scientific) and allowed to solidify at room temperature for 2 h. Then, cells suspended in complete DMEM media containing 0.3% agarose were added to the dishes preloaded with the 0.6% agarose layer. PANC-1 and BxPC-3 stably expressing control or PRDM16 gRNA were plated at a density of 1,000 cells per dish and grown for ∼2 mo. Within this time frame, PANC-1 isogenic cell lines developed small but similar colonies in size, whereas neither of the BXPC-3 isogenic cell lines developed colonies. Colonies were visualized and counted using an Olympus CKX53 microscope with the UPlanFL N 4×/0.13 iPC objective.

For the cell proliferation assay, isogenic PANC-1 (50,000 cells/well) and BxPC-3 (100,000 cells/well) cell lines stably expressing control or PRDM16 gRNA were inoculated into 6-well plates. Three (for PANC-1) and six (for BxPC-3) days after inoculation, cells were trypsinized and mixed with equal volumes of trypan blue (#T10282; Invitrogen) before being counted using an automatic cell counter (#AMQAF2000; Invitrogen Countess 3 FL). Each well was counted twice and averaged to ensure accurate cell counts were obtained.

For the in vivo growth assay, NOD scid gamma (NSG) mice were injected subcutaneously with isogenic PANC-1 and BxPC-3 cell lines stably expressing control or PRDM16 gRNA (106 cells) under septic conditions. During the observation period of ∼2 mo, mice were maintained in sterile conditions and sacrificed if they displayed any symptoms of illness. At the end of the observation period, tumors were dissected, weighted, and imaged using a 12-megapixel f/1.8 aperture camera.

### Mice

NOD scid gamma (NSG), Prdm16fl/fl, Smad4fl/fl, TβR2fl/fl and Trp53fl/fl mice were obtained from Jackson Laboratories. Loxp-Stop-Loxp-KrasG12D (LSL-KrasG12D) and Pdx1-Cre mice were obtained from the NCI Mouse Repository. p16Ink4A-Luciferase (p16Luc) was kindly provided by Dr. Sharpless (Burd et al., 2013). All PDAC mouse models were generated through successive crossbreeding of Prdm16fl/fl, Smad4fl/fl, TβR2fl/fl, p16Luc, Trp53fl/fl, LSL-KrasG12D and Pdx1-Cre mice as appropriate. Full descriptions of the genotypes of mice used throughout the study are: KC: LSL-KrasG12D;Pdx1-Cre; Prdm16KO: Prdm16fl/fl;Pdx1-Cre; KPrC: LSL-KrasG12D;Prdm16fl/fl;Pdx1-Cre; KSC: LSL-KrasG12D;Smad4fl/fl;Pdx1-Cre; KSPrC: LSL-KrasG12D;Smad4fl/fl;Prdm16fl/fl;Pdx1-Cre; KPC: LSL-KrasG12D;LSL-Trp53fl/fl;Pdx1-Cre; KIC: LSL-KrasG12D;p16Luc+/+;Pdx1-Cre; KTβC: LSL-KrasG12D;TβR2fl/fl;Pdx1-Cre.

The Institutional Animal Care and Use Committee (IACUC) of the University of Mississippi Medical Center (UMMC) or Virginia Commonwealth University (VCU) approved all animal experiments. All experiments with transgenic mouse models (including KSPrC mice) were initiated at UMMC and continued at VCU. We did not see any significant difference in the onset of tumor formation or survival in mice generated or maintained in both sites.

All mice were maintained on a mixed C57BL/6 and FVB/N genetic background. Mice were maintained in twelve-hour light/dark cycles (6:00 AM–6:00 PM) at 22°C and fed a standard rodent chow diet. Formation of PDAC in all mice enrolled in the study was confirmed using pancreatic tissue sections stained with hematoxylin and eosin (H&E) or immunostained with an anti-cytokeratin 19 antibody. Blood glucose levels were measured with blood collected from the tail vein using the ReliON Prime blood glucose strips. The average of one measurement from 2 to 3 different blood ReliON meters was used for each mouse.

### Clinical samples

Human tissue micro arrays for pancreatic tissues (#PA242b, *n* = 24; #PA483c, *n* = 48; #PA805c, *n* = 80) were purchased from US Biomax, Inc.

### Kaplan-Meier survival analysis in patients with wild-type or mutant SMAD4

In order to compare the survival of patients with high versus low expression of PRDM16 in the context of wild-type or mutated SMAD4, PRDM16 expression data (mRNA expression z-scores relative to all samples (log RNA Seq V2 RSEM) were first downloaded from the TCGA PanCancer Atlas in cBioPortal. Then, patients of the TCGA-PAAD cohort with wild-type (*n* = 140) or mutated (*n* = 26) SMAD4 were identified using the COSMIC database. Next, patients were classified as having high or low PRDM16 expression based on whether they were above or below the top and bottom quartile of PRDM16 expression of the TCGA-PAAD cohort, respectively. Lastly, each patient was matched with the corresponding expression of PRDM16 and SMAD4 mutational status as well as the time to death or to last follow up (depending on their vital status) to create a Kaplan-Meier survival curve.

### PRDM16 expression in patients with or without SMAD4 mutations

To assess the expression of PRDM16 in patients in the context of wild-type or mutated SMAD4, PRDM16 expression data (mRNA expression z-scores relative to all samples, log RNA Seq V2 RSEM) were first downloaded from the TCGA PanCancer Atlas in cBioPortal. Then, patients of the TCGA-PAAD cohort with different types of SMAD4 mutations were identified using the cBioPortal interface. Patients were then filtered based on those with no alteration or with truncating mutations in SMAD4 in order to create a violin plot comparing the normalized PRDM16 expression between these two groups.

### qRT-PCR

Total RNA was extracted from frozen mouse tissue samples using TRIzol (#15596018; Ambion) and purified with chloroform (#066903; Thermo Fisher Scientific) and ethanol (#BP2818; Thermo Fisher Scientific). The RNA was then reverse-transcribed using a High-Capacity cDNA Reverse Transcription kit (#4368814; Applied Biosystems). The cDNA product was analyzed by qRT-PCR. Briefly, 25 ng cDNA and 150 nmol of each primer were mixed together with the SsoFast EvaGreen Supermix (#1725200; BioRad). PCR reactions were conducted using a CFX96 Real-Time System (BioRad) in a 96-well plate. The relative mRNA levels were calculated with the comparative CT method and normalized to GAPDH mRNA.

#### Primers used for human samples

PRDM16-For 5′-CTT​TGA​CCA​CAC​CCG​AAG​GT-3′; PRDM16-Rev 5′-TGT​GGA​GAG​GAG​TGT​CTT​CG-3′; JUNB-For 5′-CCT​GGA​CGA​TCT​GCA​CAA​GA-3′; JUNB-Rev 5′-GGT​TGG​TGT​AAA​CGG​GAG​GT-3′; GAPDH-For 5′-CCA​TGG​GGA​AGG​TGA​AGG​TC-3′; GAPDH-Rev 5′-AGT​GAT​GGC​ATG​GAC​TGT​GG-3′.

#### Primers used for mouse samples

Prdm16-For 5′-TCC​CAC​CAG​ACT​TCG​AGC​TA-3′; Prdm16-Rev 5′-AAA​GTC​GGC​CTC​CTT​CAG​TG-3′; Gapdh-For 5′-CAC​CAT​CTT​CCA​GGA​GCG​AG-3′; Gapdh-Rev 5′-CAC​CAT​CTT​CCA​GGA​GCG​AG-3′.

### Chromatin immunoprecipitation assay (ChIP)

ChIP assays were performed using a kit following the manufacturer’s instructions (#17-295; Millipore). Accordingly, cells were first treated with 1% formaldehyde and incubated at 37°C for 10 min. Next, cells were washed twice with ice-cold PBS containing protease inhibitors. Cells were then scraped and pelleted by centrifugation at 2,000 RPM for 4 min at 4°C. Then, cells were resuspended in SDS Lysis Buffer (Millipore, #20-163) and incubated for 10 min on ice. After samples were centrifuged for 10 min at 13,000 RPM at 4°C, the supernatants were diluted 10 times by adding ChIP Dilution Buffer (#20-153; Millipore) containing protease inhibitors. The diluted supernatants were then treated with 75 μl of a 50% slurry of Protein-A Agarose/Salmon Sperm DNA (#16-157C; Millipore) at 4°C for 30 min with agitation. After centrifugation, supernatants were immunoprecipitated with antibodies against Smad4, Smad2, Smad3, Prdm16, GAPDH or isotype-matched control IgG and 60 μl of a 50% slurry of Protein-A Agarose/Salmon Sperm DNA at 4°C for 1 h with rotation. Agarose was pelleted using centrifugation at 1,000 RPM for 1 min at 4°C. The pellets were washed for 5 min in Low Salt Immune Complex Wash Buffer (#20-154; Millipore) once, High Salt Immune Complex Wash Buffer (#20-155; Millipore) once, LiCl Immune Complex Wash Buffer (#20-156; Millipore) once, and TE Buffer (#20-157; Millipore) twice. To amplify DNA bound to the immunoprecipitates, elution buffer (1% SDS, 0.1 M NaHCO_3_) was added to each sample followed by agitation and incubation for 15 min with rotation at room temperature. Eluates were then mixed with NaCl (final concentration of 0.2 M) and incubated for 4 h at 65°C followed by adding EDTA (0.01 M), Tris-HCl, pH 6.5 (0.04 M), and Proteinase K (0.04 mg/ml). Samples were then incubated for 1 h at 45°C, and DNA was recovered using phenol/chloroform extraction coupled with ethanol precipitation. Pellets were washed with 70% ethanol and air-dried. Lastly, pellets were resuspended in an appropriate buffer for PCR, and PCR products were analyzed on a 2% agarose gel. The immunoprecipitated DNA was also analyzed by qPCR using locus specific primers and normalized to input DNA. Relative fold enrichment in each locus was quantified relative to the control as described above (qRT-PCR) as well as in our published studies (Parajuli et al., 2018; Zhang et al., 2015). The following primers were used: PRDM16-For 5′-CAT​CTC​CCC​AGC​ATT​GTC​AGT-3′; PRDM16-Rev 5′-GGA​GCG​CCG​AAC​ACG​GAA​TG-3′; JUNB-For 5′-GGC​AAA​GCC​CAG​GGT​CAA​TA-3′; JUNB-Rev 5′-AAA​GCT​AGT​AAG​CGG​CCT​GG-3′; GAPDH-For 5′-CGG​GAT​TGT​CTG​CCC​TAA​TTA​T-3′; GAPDH-Rev 5′-GCA​CGG​AAG​GTC​ACG​ATG​T-3′.

### Luciferase reporter assay

PANC-1 cells were plated in 6-well plates and transfected with the CAGA9-Lux or Prdm16-Lux reporter in the presence of pcDNA3.1-Prdm16, pCMV5-HA-Smad4, or empty vector (pcDNA3.1 or pCMV5-HA as appropriate) using X-tremeGENE9 (#0635779001; Sigma-Aldrich). The pRL-SV40 plasmid (#AF025845; Promega) was cotransfected to normalize for transfection efficiency. For CAGA9-Lux assays, cells were incubated for 24 h with the transfection mixtures and then treated with 5 ng/ml TGF-β1 (#7754-BH; R&D Systems) for 24 h before measuring luciferase activity using the Dual-Luciferase Reporter Assay System (#E1910; Promega). For Prdm16-Lux assays, cells were incubated for 48 h with the transfection mixtures and then processed for luciferase activity as described for CAGA9-Lux. Firefly Luciferase activity was normalized based on Renilla luciferase expressed from pRL-SV40 plasmid.

### Co-immunoprecipitation

Cell lysates were prepared in lysis buffer (25 mM Tris-HCl, pH 7.2, 150 mM NaCl, 5 mM MgCl_2_, 5% glycerol and 1% NP40) supplemented with phosphatase inhibitors (#P5726; Sigma-Aldrich) and EDTA-free protease inhibitors (#P8340; Sigma-Aldrich). Cells were lysed with 1 ml of lysis buffer for 10 min on ice and protein concentrations were determined using the BCA reagent (#23227; Thermo Fisher Scientific). Then, 90% of the pre-cleared lysates were added to anti-Smad4 antibody for 1 h at 4°C under constant rocking, and then protein A magnetic beads (#G8781; Promega) were added for an additional 1 h at 4°C. The beads were subsequently pelleted and washed five times with lysis buffer and eluted for immunoblotting using 1X SDS-PAGE sample buffer (#NP0007; Thermo Fisher Scientific). The other remaining 10% of lysate was used to determine total protein levels by direct immunoblotting.

### Immunoblotting

Cell extracts were prepared in lysis buffer containing 20 mM Tris HCl (pH 7.5), 150 mM NaCl, 1 mM EDTA, 1 mM EGTA, 1% Triton, 2.5% sodium pyrophosphate, 1 mM β-glycerophosphate, 1 mM Na_3_VO_4_, 1 µg/µL leupeptin, protease inhibitors (#P8340; Sigma-Aldrich) and phosphatase inhibitors (#P5726; Sigma-Aldrich). Protein concentrations were determined using the BCA reagent as described earlier, and samples were denatured using SDS sample buffer (#1610747; BioRad). Samples were loaded into a Criterion Tris-Glycine Extended Gel (#5671124; BioRad) and separated by electrophoreses at 60 mA. The gels were then transferred onto a nitrocellulose membrane (#1620115; BioRad) by a wet transfer system (BioRad) at 100V for 1 h at room temperature. All membranes were then blocked by incubation with 5% dry milk in TBST (TBS with 0.1% Tween20) for 1 h at room temperature. Membranes were probed with the primary antibody overnight at 4°C in the blocking buffer, washed with TBST, and incubated with the peroxidase-conjugated secondary antibody. Enhanced chemiluminescence (ECL) Western blotting substrates (#170-5061; BioRad) were used for the visualization of the results. The acquisition of images was performed using the ChemiDoc MP Imaging System (BioRad).

### Histology, immunohistochemistry, and immunofluorescence

Tissue samples were fixed in 10% formalin and embedded in paraffin. For pancreatic tissue histology, paraffin sections were stained with hematoxylin and eosin (H&E) using standard techniques. Briefly, sections were deparaffinized with xylene and rehydrated in a graded series of ethanol. They were then successively immersed in a hematoxylin solution (HHS128-4L; Sigma-Aldrich) for 2 min, a clarifier solution (7402L; Epredia) for 15 s, and blueing reagent solution (7301L; Epredia) for 1 min. Between each of the three steps, sections were immersed in water for 1 min. Next, slides were immersed in an eosin solution (HT110280-2.5L; Sigma-Aldrich) for 3 min before being dehydrated 3 times for 3 min in 100% ethanol (89370-088; VWR) followed by xylene (V1001; Koptec). For immunofluorescence and immunohistochemistry, tissue sections were deparaffinized with xylene and rehydrated in a graded series of ethanol. Antigen retrieval was performed for 30 min at high temperature in citrate buffer. Then, slides were blocked and incubated overnight with anti-insulin, anti-glucagon, anti-Prdm16, anti-Muc5AC, anti-chromogranin-A, anti-αSMA, anti-E-cadherin, anti-vimentin or IgG-matched isotype control antibody (negative control) at 4°C. For immunofluorescence, slides were incubated with the secondary antibodies conjugated to Alexa-Fluor568 (#A11011; Invitrogen) or Alex-Fluor488 (#A11088; Invitrogen), co-stained with DAPI (#H1800; Vector Laboratories), and viewed on a Nikon Ti-E fluorescence microscope. Immunohistochemistry was done with the VECTASTAIN Elite ABC HRP kit (rabbit, #PK6101 or mouse, #PK-6102; Vector Laboratories) as per manufacturer’s instructions. Tissue sections were incubated for 30 min in the secondary antibody followed by the VECTASTAIN ABC reagent. Color development was done with the DAB Peroxidase Substrate kit (#SK-4100; Vector Laboratories) with or without Nickel added enhancement as appropriate.

To quantify Prdm16 expression in human samples, the TMA was scanned using the PlanApo 40 × 0.95/0.25–0.17 mm objective on the Keyence BZ-X810 automated microscope and characterized using the BZ-X800 Analyzer software from Keyence. The expression intensity of six images of normal areas and PanIN stages 1, 2, 3 and PDAC lesions were chosen in a random manner. The intensity of Prdm16 expression was obtained automatically using the BZ-X800 Analyzer software from Keyence and the means of each stage (normal, PanIN stage 1, 2, 3, PDAC) were calculated. Each area/lesion was individually quantified using the area directly around the lesion.

To quantify Prdm16 expression in mouse tissues, slides chosen in a random manner from all mice under study were scanned using the PlanApo 10 × 0.45/4.00 mm objective on the Keyence BZ-X810 automated microscope and characterized using the BZ-X800 Analyzer software from Keyence. Random images of PanIN and PDAC lesions were taken and quantified only using the area directly around the lesions. Each lesion was individually quantified and the mean ± SEM of six independent lesions was presented in figures.

The quantifications of Alcian blue staining or CK19, Muc5AC, and α-SMA immunostaining were conducted by first taking images of six normal areas or PanIN/PDAC lesions from all mice under study in a random manner using the PlanApo 40 × 0.95/0.25–0.17 mm objective on the Keyence BZ-X800 microscope. We then individually quantified each image using the Keyence BZ-X800 analyzer software from Keyence. Lastly, the mean ± SEM was calculated for each genotype.

To quantify the distribution of PDAC lesions, mouse tissue slides were scanned using the PlanApo 10 × 0.45/4.00 mm objective on the Keyence BZ-X810 automated microscope and characterized using the BZ-X800 Analyzer software from Keyence. PanIN and IPMN lesions were counted and characterized as either PanIN or IPMN. The surface area for each lesion was obtained from the Keyence software and the mean sum of the surface area for all PanIN or IPMN lesions were calculated for each genotype, including all mice recruited. The percentage of stroma was identified using the BZ-X800 Analyzer software from Keyence. The distribution of PDAC lesions was then calculated by multiplying the percentage of PanIN surface area divided by the total non-PDAC surface area of the tissue. The same process was repeated for IPMN lesions.

All images were taken using the Zeiss Axio Lab.A1 upright (Zeiss EC Plan-NEOFLUAR 40×/0.9 Pol and Zeiss A-Plan 10×/0.25 objectives), Zeiss Observer.A1 inverted (Zeiss LDA-Plan 40×/0.55 Ph1 objective), or Leica DM1000LED upright microscopes (Leica HI PLAN 40×/0.65 objective). The numerical aperture of the objective lenses are 0.9 and 0.25, 0.55, and 0.65, respectively, with a temperature of 1 (10 Kelvin) with an imaging medium of air. The fluorochromes analyzed in immunofluorescence experiments were were Alexa-Fluor568 (red), Alexa-Fluor488 (green) and DAPI (blue). The cameras used were the Axiocam ICc5, Axiocam503mono, and LeicaDM2900 with the acquisition software was ZEN 2 lite for both Zeiss microscopes and LAS X for the Leica microscope. Subsequent software used for incorporating images into figures was Adobe Photoshop followed Microsoft PowerPoint.

### Statistical analysis

The values are expressed as mean ± SEM. The error bars (SEM) shown for all results were derived from biological replicates, not technical replicates. Significant differences between two groups were evaluated using either a two-tailed, unpaired Mann–Whitney test or two-tailed, unpaired *t* test, which was found to be appropriate for the statistics, as the sample groups displayed a normal distribution and comparable variance. Statistical significance of survival differences was determined by log-rank test.

### Online supplemental material

Fig. S1 shows that Prdm16 is transiently expressed in the premalignant lesions. Fig. S2 shows that Prdm16KO mice display normal insulin and glucagon expression and distribution as well as normal blood glucose levels. Fig. S3 provides additional data demonstrating that inactivation of Prdm16 accelerates KrasG12D-driven PDAC. Fig. S4 displays that Prdm16 is required for IPMN-to-PDAC progression and that Prdm16 deletion led to the accumulation of cells with high vimentin expression. In addition, Fig. S4 shows the effects of deleting PRDM16 on the proliferation of PANC-1 and BxPC-3 cell lines. Fig. S5 further expands upon the notion that concomitant inactivation of Prdm16 and Smad4 mimics the phenotype of complete TGF-β signaling inactivation through TβRII ablation.

## Supplementary Material

SourceData F5 is the source file for Fig. 5.

SourceData F6 is the source file for Fig. 6.
