# Supplementary figures and images for: Antagonism between Prdm16 and Smad4 specifies the trajectory and progression of pancreatic cancer
Source: J Cell Biol. 2023 Feb 24;222(4):e202203036. doi: 10.1083/jcb.202203036 (PMC9999015; doi:10.1083/jcb.202203036)

Figure 5D

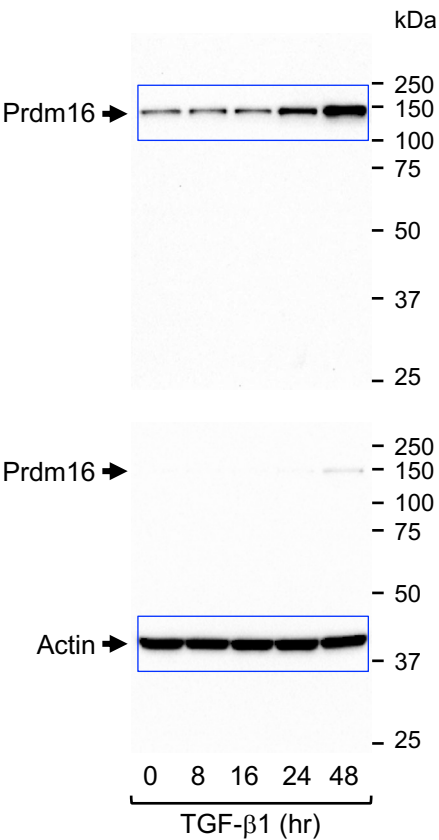

Supplement: SourceData F5 — is the source file for Fig. 5. [file JCB_202203036_SourceDataF5.pdf]

Figure 6A

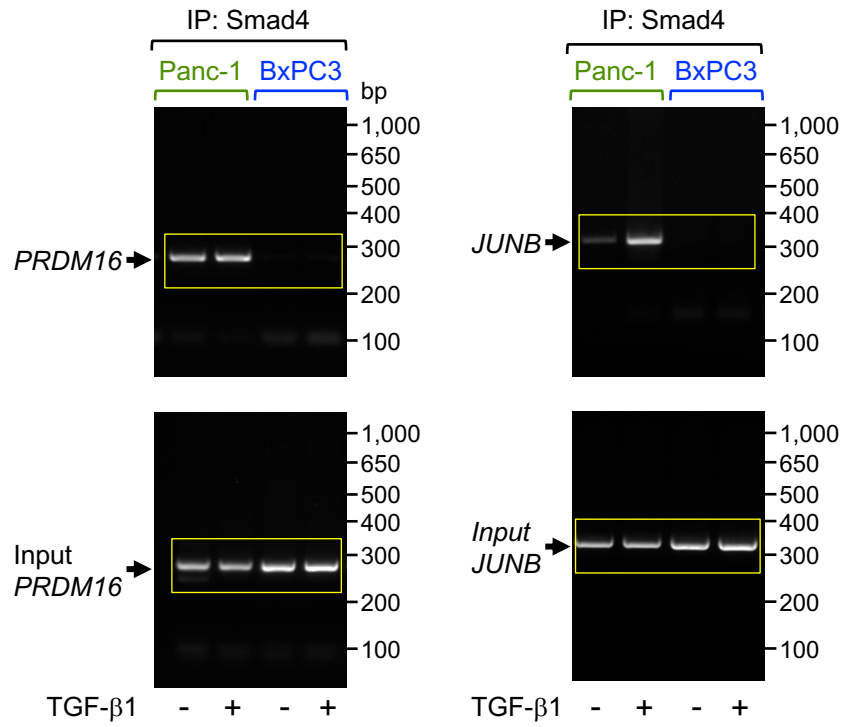

Figure 6B

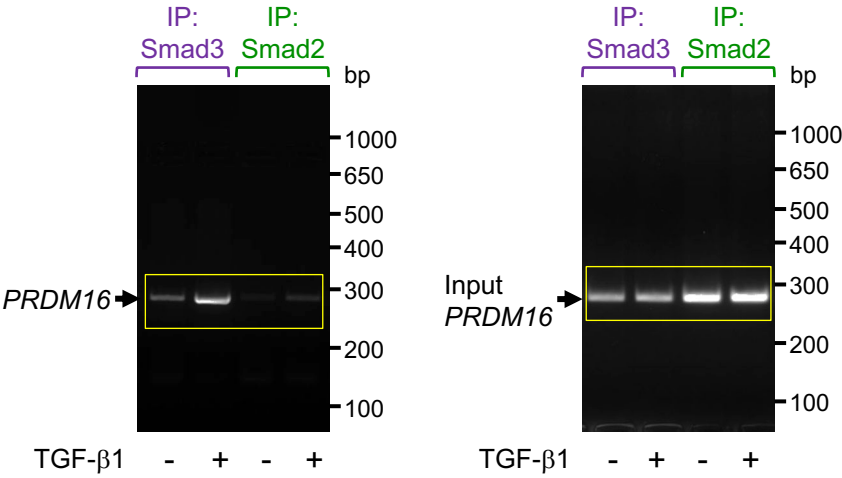

Figure 6C

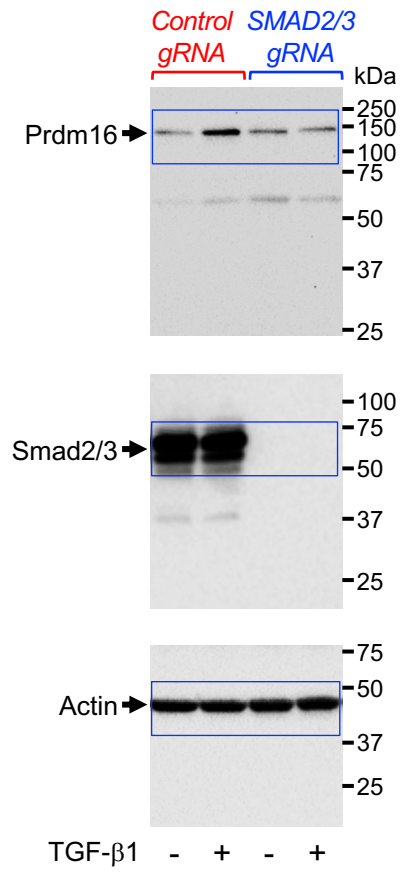

Figure 6E

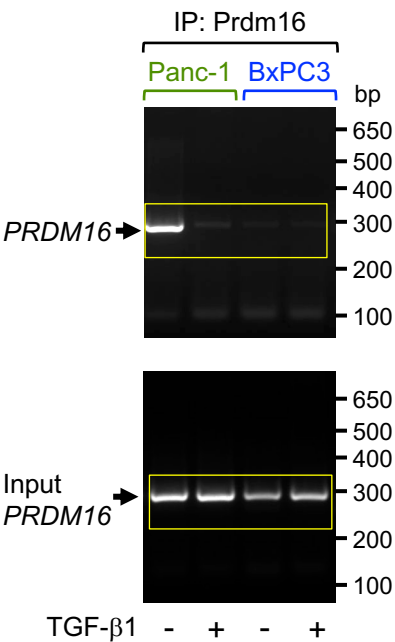

Figure 6F

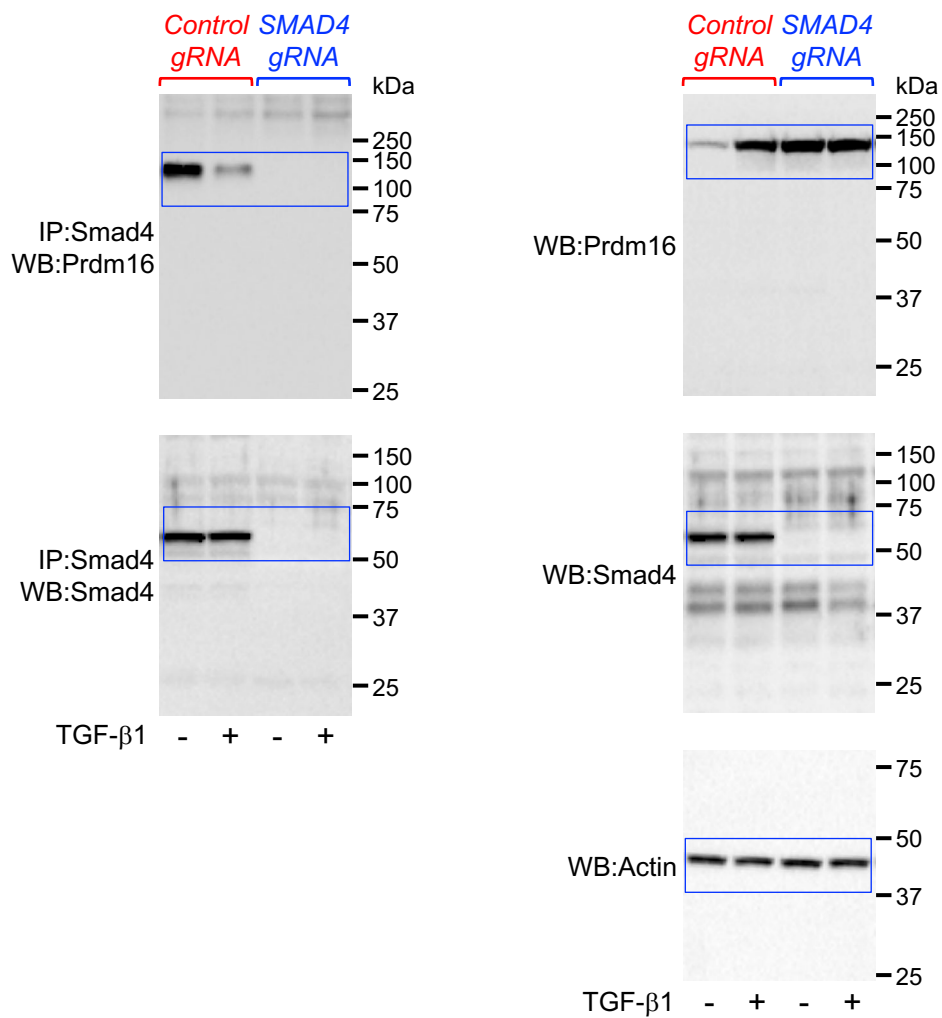

Supplement: SourceData F6 — is the source file for Fig. 6. [file JCB_202203036_SourceDataF6.pdf]
